# Dual roles of TRIM3 in colorectal cancer by retaining p53 in the cytoplasm to decrease its nuclear expression

**DOI:** 10.1038/s41420-023-01386-1

**Published:** 2023-03-09

**Authors:** Yang Han, Su Lu, Chenlong Song, Yi Xuan, Meng Zhang, Hong Cai

**Affiliations:** 1 Department of Gastric Surgery, Fudan University Shanghai Cancer Center, Shanghai, 200032 China; 2 Department of Oncology, Shanghai Medical College, Fudan University, Shanghai, 200032 China; 3 Department of Pathology, Shanghai General Hospital, School of Medicine, Shanghai Jiao Tong University, Shanghai, 20080 China; 4 Department of General Surgery, Shanghai General Hospital, School of Medicine, Shanghai Jiao Tong University, Shanghai, 20080 China; 5 Department of Pathology, Fudan University Shanghai Cancer Center, Shanghai, 200032 China

**Keywords:** Colorectal cancer, Cancer epigenetics

## Abstract

Colorectal cancer is a very heterogeneous disease caused by the interaction of genetic and environmental factors. P53, as a frequent mutation gene, plays a critical role in the adenoma-carcinoma transition during the tumorous pathological process. Our team discovered TRIM3 as a tumor-associated gene in CRC by high-content screening techniques. TRIM3 demonstrated both tumor-suppressive and tumorigenic features in cell experiments dependent on the cell status of wild or mutant p53. TRIM3 could directly interact with the C terminus of p53 (residues 320 to 393), a common segment of wtp53 and mutp53. Moreover, TRIM3 could exert different neoplastic features by retaining p53 in the cytoplasm to decrease its nuclear expression in a wtp53 or mutp53-dependent pathway. Chemotherapy resistance develops in nearly all patients with advanced CRC and seriously limits the therapeutic efficacies of anticancer drugs. TRIM3 could reverse the chemotherapy resistance of oxaliplatin in mutp53 CRC cells by degradation of mutp53 in the nuclei to downregulate the multidrug resistance gene. Therefore, TRIM3 could be a potential therapeutic strategy to improve the survival of CRC patients with mutp53.

## Introduction

Colorectal cancer (CRC) is the third most common cancer and the fourth leading cause of cancer death in the world [1, 2]. Although early detection and perioperative treatment have improved survival, advanced diseases remain incurable [3, 4]. CRC is a very heterogeneous disease caused by the interaction of genetic and environmental factors, except for a few mutations of the APC, k-Ras, and p53 genes. It is thought that p53 mutation plays a critical role in the adenoma-carcinoma transition during the tumorous pathological process [5–7]. The frequency of p53 mutation in CRC is approximately 40% to 50% [8]. Different types of p53 mutation could determine differently biologic behaviors of CRC, such as invasive depth, metastatic site, and even poor prognosis [5, 8]. P53 mutation can demonstrate abnormal gain-of-functions (GOF) to facilitate oncogenesis, metastasis, and chemoresistance [9]. Therefore, restoration wild-type function of mutant p53 (mutp53) or inactivation of mutp53 has a great potential as a novel therapeutic strategy for CRC. However, the majority of molecules capable of making mutp53 loss of function or inhibition expression have only been tested in cell lines and animal models, there is no good solution existed in clinical trials [6, 10–14].

Our team designed and prepared an RNA interference library targeting a large number of genes in CRC previously. We screened a series of genes expression after RNA interference by high-content screening (HCS) techniques [15]. We discovered that TRIM3 (Tripartite-motif containing 3) knockdown could significantly inhibit the proliferation of RKO cells. TRIM3 is a member of the TRIM(Tripartite-motif) protein superfamily implicated in a wide range of physiologic processes such as immunity, proliferation, oncogenesis, and transcriptional regulation [16, 17]. TRIM3 is highly enriched in neurons and reported to abnormally express in several human cancers, including glioblastoma and hepatocellular carcinoma [18–22]. However, the detailed expression and mechanism of TRIM3 in CRC have remained unknown to date.

In the present study, we discovered TRIM3 exhibited both pro-tumorigenic and tumor-suppressive features in cell experiments dependent on the cell status of wild or mutant p53. TRIM3 could directly interact with the C terminus of p53, a common segment of wild type p53 (wtp53) and mutp53, to decrease p53 in the nuclei by retaining them in the cytoplasm. The reduced p53 could exert a pro-tumorigenic or tumor-suppressive role of colorectal carcinogenesis in a wtp53 or mutp53-dependent pathway. Moreover, TRIM3 could reverse the sensitivity to oxaliplatin in mutp53 CRC cells by downregulating the multidrug resistance gene (MDR1). Therefore, TRIM3 could be a potential therapeutic strategy to improve the survival of CRC patients with mutp53 by degradation of mutp53 in the nuclei.

## Results

### Knockdown TRIM3 significantly inhibited RKO cell proliferation

**Fig. 1:**
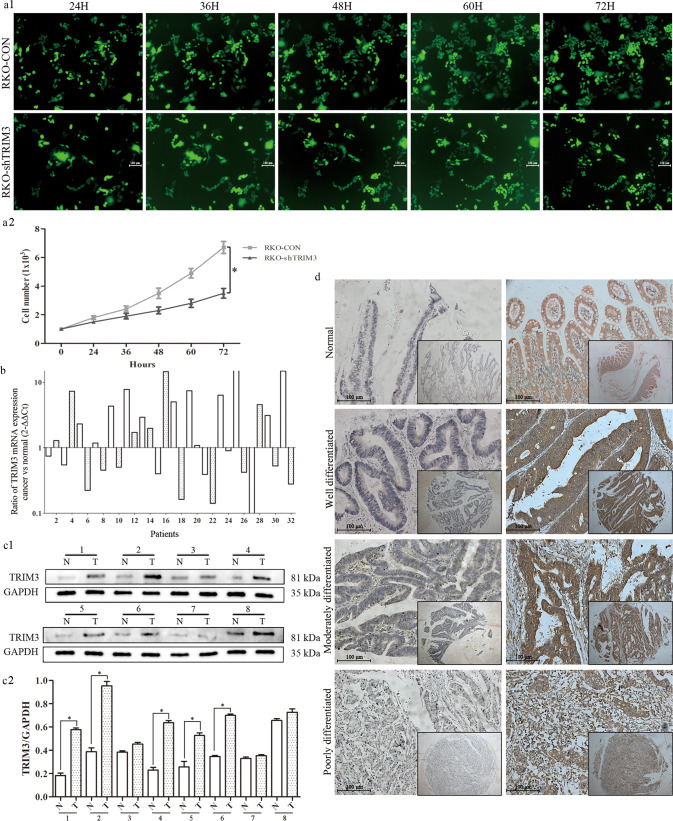
RKO cells with TRIM3 knockdown monitored by HCS technology and TRIM3 expression in CRC tissues. **a1** Dynamic growth of GFP-labeled RKO cells transfected with control and TRIM3-shRNA was monitored twice a day for 3 consecutive days by HCS. (Magnification ×50); **a2** The growth curves of RKO cells transfected with control and TRIM3-shRNA at different time points; **b** TRIM3 mRNA expressions in 32 pairs of CRC samples. white histogram: odd samples; gray histogram: even samples; **c1** TRIM3 protein expression in representative eight pairs of CRC samples (T) and adjacent non-cancerous mucosa (N) by Western Blot; **c2** The quantification expression of TRIM3 in eight pairs of CRC samples; **d** Immunohistochemical staining of TRIM3 in CRC tissue microarray. Original magnification ×50 and ×200 for inset images.

Our team designed and prepared an RNA interference library targeting a series of genes associated with CRC globally by HCS. RKO cell, as a common human colon cancer cell model, was used to transfect and screen their cellular proliferation abilities. The dynamic growth of cells with GFP-labeled was monitored twice a day for 3 consecutive days using HCS (Fig. 1a). As illustrated in Fig. 1a, the number of GFP-labeled cells in the TRIM3-shRNA group was greatly decreased compared to the control group in a time-dependent manner.

### The expression of TRIM3 was slightly elevated in CRC tissues

Among the 32 paired cancerous and adjacent normal mucosal samples, TRIM3 mRNA expression was elevated in 56.3% (18/32) CRC tissues, compared to matched adjacent normal mucosa (Fig. 1b). Subsequent western blot analysis demonstrated that TRIM3 protein levels were higher in cancerous tissues but not obvious (Fig. 1c). Moreover, immunohistochemical staining indicated that 29% (101/348) were TRIM3 positive expression of the 348 adjacent non-cancerous mucosa samples in the paired TMA. In contrast, among the 348 CRC specimens, 58% (202/348) were TRIM3 positive and nearly half were negative expression (Fig. 1d, Table 1).

**Table 1:** Expression of TRIM3 and P53 in adjacent non-cancerous mucosa and cancer tissues.

	*N*	TRIM3 expression		*P*-value	P53 expression		*P*-value
		Negative (%)	Positive (%)		Negative (%)	Positive (%)	
Adjacent mucosa	348	247 (71%)	101 (29%)	<0.001*	303 (87.1%)	45 (12.9%)	<0.001*
Cancer tissues	348	146 (42%)	202 (58%)		136 (39.1%)	212 (60.9%)	

*P*-value are based on chi-squared.

*Significant difference.

### TRIM3 displayed both pro-tumorigenic and tumor-suppressive features in cell experiments dependent on the cell status of wild or mutant p53

**Fig. 2:**
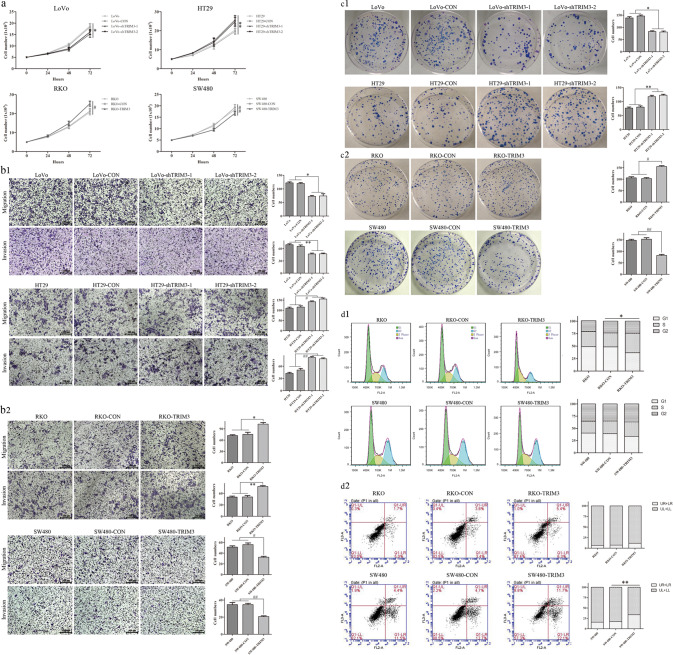
The dual neoplastic features of TRIM3 related with CRC cells status of wild or mutant p53. **a** The growth curves of CRC cells with TRIM3 knockdown or overexpression by CCK8; **b1** The number of cell migration and invasion of CRC cells with TRIM3 knockdown; **b2** The number of cell migration and invasion of CRC cells with TRIM3 overexpression; **c1** The number of colony formation of CRC cells with TRIM3 knockdown; **c2** The number of colony formation of CRC cells with TRIM3 overexpression; **d1** The effects of TRIM3 overexpression on cell cycle in RKO and SW480 cells by flow cytometry; **d2** The effects of TRIM3 overexpression on cell apoptosis in RKO and SW480 cells by flow cytometry; wtp53 cells: LoVo, RKO; mutp53 cells: SW480, HT29; All data were obtained from three independent experiments, and expressed as mean ± standard deviation. *, ** and ^#^,^##^*P* < 0.05 in comparison with control groups.

To investigate the role of TRIM3 in CRC progression, we explored the capacities of proliferative, invasive, and migratory in CRC cells after TRIM3 knockdown or overexpression. The selection of CRC cell lines was based on TRIM3 expression (Supplementary Fig. 1) (wtp53 cells: LoVo, RKO; mutp53 cells: SW480, HT29). After TRIM3 was knocked down in LoVo cells (wtp53 status), the growth curves of TRIM3-shRNA cells at the time points of 48 and 72 h were significantly reduced compared with the control group (Fig. 2a). The number of cells across the membrane in the matrigel-free and matrigel chamber in the TRIM3-shRNA group was significantly lower in comparison to the control group (Fig. 2b1). Meanwhile, the number and size of colonies in the TRIM3-shRNA group displayed a dramatic decrease of 2-fold relative to the control group (Fig. 2c1). However, when TRIM3 was inhibited in HT29 cells (mutp53 status), the abilities of cell viability, migration, invasion, and colony formation were significantly increased compared with the control group, which was opposed to the results from LoVo cells (wtp53 status) (Fig. 2a/b1/c1). When TRIM3 was overexpressed in RKO cells (wtp53 status), the abilities of cell proliferation, colony formation, migration, and invasion were obviously increased in comparison with the empty vector-transfected control group (Fig. 2a/b2/c2). However, after TRIM3 was overexpressed in SW480 cells (mutp53 status), the abilities of cell proliferation, migration, invasion, and colony formation were significantly weakened, which was opposed to the results from RKO cells (wtp53 status) (Fig. 2a/b2/c2).

Moreover, TRIM3 overexpression in RKO cells (wtp53 status) was associated with the cell cycle. The proportions of RKO cells with TRIM3 overexpression in the G0/ G1 phase of the cell cycle were obviously decreased compared with the control group (Fig. 2d1). Meanwhile, TRIM3 overexpression in SW480 cells (mutp53 status) was related to cell apoptosis. The rates of apoptosis in SW480 cells with TRIM3 overexpression were significantly increased than the control group (Fig. 2d2).

Therefore, there seemed to be a relationship between TRIM3 neoplastic features and p53 status (wild or mutant type). It appeared that TRIM3 overexpression in wtp53 cells (LoVo and RKO) promoted cancer and arrested cell cycle, whereas overexpression of TRIM3 in mutp53 cells (HT29 and SW480) suppressed cancer and promoted cell apoptosis.

### TRIM3 close correlation with p53 in expression and direct binding with p53 at the C terminus

**Fig. 3:**
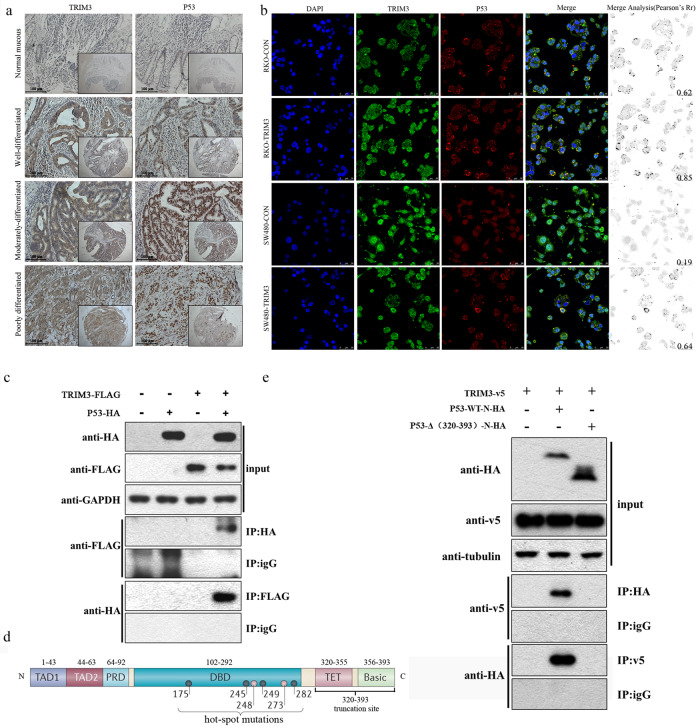
TRIM3 close correlation with p53 in expression and direct binding with p53 at the C terminus. **a** The expression and relationship between TRIM3 and p53 in the identical area of different differentiated cancer by immunohistochemistry. Original magnification ×50 and ×200 for inset images; **b** TRIM3 and p53 co-staining as distinct dots in the cytoplasm of RKO and SW480 cells by confocal microscopy and Image J software; **c** TRIM3 direct binding with p53. 293 T cells were transfected with plasmids encoding TRIM3-FLAG and P53-HA or empty vectors for 24 h, followed by Co-IP and a western blotting analysis with anti-HA, anti-GAPDH, and anti-FLAG antibodies; **d** The diagram of p53 hot-spot mutation sites and our designed truncation site; **e** TRIM3 binding to the C terminus of p53 (residues 320 to 393), a common segment of wild and mutant p53. HA-tagged wtp53 and C-terminal (320–393) truncation mutp53 were transfected into 293 T cells expressing v5-TRIM3. After the beads were washed, bound proteins were eluted and analyzed by Western Blot with anti-HA, anti-tubulin, and anti-v5 antibodies.

The phenomenon that TRIM3 pro-tumorigenic or tumor-suppressive features were closely related to p53 wild or mutant status led us to explore the relationship between TRIM3 and p53. As shown in Tables 1 and 2, of the 348 paired TMA, p53 expressions were obviously up-regulated in cancerous tissues compared with those in the corresponding non-cancerous mucosa (*P* < 0.001). Of note, TRIM3 expressions were significantly correlated with p53 expression in clinicopathological parameters (*P* < 0.001). The expression trends of TRIM3 and p53 were fundamentally consistent in the identical area of the same tumor tissues, as demonstrated by immunohistochemistry (Fig. 3a). Moreover, confocal microscopy was conducted to analyze the localization of TRIM3-p53 co-staining. TRIM3 was located in the cytoplasm. P53 was predominantly throughout the nuclei, partly in the cytoplasm (Fig. 3b). TRIM3 and p53 displayed co-staining as distinct punctate in the cytoplasm of RKO and SW480 cells with TRIM3 overexpression (Fig. 3b). In addition, with Flag-tagged TRIM3 coprecipitation with HA-tagged p53 from 293 T cell lysates, TRIM3 was discovered to interact with p53 directly (Fig. 3c). Furthermore, HA-tagged wtp53 and C-terminal (320-393) truncation mutp53 were constructed and transfected into 293 T cells expressing v5-TRIM3 (Fig. 3d). The results indicated that TRIM3 interacted directly with the C terminus of p53 (residues 320 to 393), a common segment of wild and mutant p53 (Fig. 3e).

**Table 2:** Correlation between clinicopathological features and TRIM3 expression (*N* = 348).

	*N*	TRIM3 expression		
		Negative(146)	Positive(202)	*P*
Age				0.533
<50 years	20	6 (30.0%)	14 (70.0%)	
50–75 years	207	90 (43.5%)	117 (56.5%)	
>75 years	121	50 (41.3%)	71 (58.7%)	
Sex				0.232
Male	169	65 (38.5%)	104 (61.5%)	
Female	179	81 (45.3%)	98 (54.7%)	
Tumor size				0.604
<4 cm	115	52 (45.2%)	63 (54.8%)	
4–6 cm	175	69 (39.4%)	106 (60.6%)	
>6 cm	58	25 (43.1%)	33 (56.9%)	
Tumor location				0.403
Ascending	85	34 (40.0%)	51 (60.0%)	
Transverse	16	6 (37.5%)	10 (62.5%)	
Descending	30	17 (56.7%)	13 (43.3%)	
Sigmoid	91	41 (45.1%)	50 (54.9%)	
Rectum	126	48 (38.1%)	78 (61.9%)	
T stage				0.172
T1	19	9 (47.4%)	10 (52.6%)	
T2	51	28 (54.9%)	23 (45.1%)	
T3	198	80 (40.4%)	118 (59.6%)	
T4	80	29 (36.3%)	51 (63.8%)	
N stage				0.920
N0	224	96 (42.9%)	128 (57.1%)	
N1	84	34 (40.5%)	50 (59.5%)	
N2	40	16 (40.0%)	24 (60.0%)	
M stage				0.010*
M0	324	142 (43.8%)	182 (56.2%)	
M1	24	4 (16.7%)	20 (83.3%)	
AJCC stage				0.026*
I	60	31 (51.7%)	29 (48.3%)	
II	155	63 (40.6%)	92 (59.4%)	
III	109	48 (44.0%)	61 (56.0%)	
IV	24	4 (16.7%)	20 (83.3%)	
Differentiation				0.619
Well	124	50 (40.3%)	74 (59.7%)	
Moderate	206	90 (43.7%)	116 (56.3%)	
Poorly	18	6 (33.3%)	12 (66.7%)	
P53				<0.001*
Negative	136	79 (58.1%)	57 (41.9%)	
Positive	212	67 (31.6%)	145 (68.4%)	

*P*-value are based on chi-squared or Fisher’s exact test.

*Significant difference.

### TRIM3 exerted dual neoplastic roles by retaining p53 in the cytoplasm to decrease its nuclear expression

**Fig. 4:**
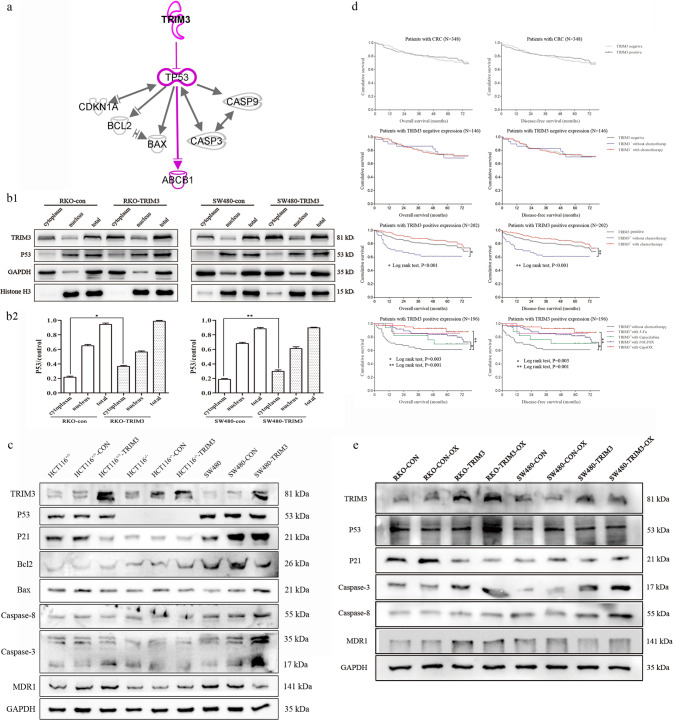
TRIM3 could alter downstream pathway of p53 and MDR1 expression by retaining p53 in the cytoplasm to decrease its nuclear expression. **a** The target genes and downstream pathway of TRIM3 and p53 predicted by IPA software. TRIM3 and p53 were used as seeds to construct their related interaction network available in the Ingenuity database. Solid lines: direct interaction; Solid lines without arrow head: molecular interaction; Solid lines with arrow head: molecular regulation; **b1** The expression of p53 in the nuclei and cytoplasm after TRIM3 overexpression; **b2** The quantification of p53 determined by densitometry of protein bands. GAPDH was the loading control in the nuclei and total; Histone H3 was the loading control in the cytoplasm; **c** The expression of downstream pathway of p53 after TRIM3 overexpression in the three p53-specific cell lines (HCT116^+/+^, HCT116^−/−^ and SW480); **d** The survival curves of CRC patients with regard to TRIM3 expression and different chemotherapy regiments; **e** The expression of downstream pathway of TRIM3 in wtp53 and mutp53 cells after treatment with oxaliplatin.

To explore the mechanisms of how TRIM3 was bound with p53 to exert dual neoplastic features, we used TRIM3 and p53 as seeds to construct their related interaction network by Ingenuity Pathway Analysis (IPA) software. The predicted target genes and downstream pathways were shown in Fig. 4a. TRIM3 could retain p53 in the cytoplasm to decrease its levels in the nuclei by direct binding with each other. The reduced nuclear expressions of p53 could influence cell cycle and apoptosis-related proteins.

To verify the prediction, we detected p53 expression in the nucleus and cytoplasm after TRIM3 overexpression. The results showed that TRIM3 overexpression in either RKO (wtp53) or SW480 cells (mutp53) could obviously increase p53 expression in the cytoplasm and decrease its expression in the nucleus, but almost made no change in total protein levels (Fig. 4b). Furthermore, to validate whether TRIM3 exerted dual neoplastic features in a wtp53 or mutp53-dependent pathway as predicted, HCT116^+/+^ (wtp53), HCT116^−/−^ (p53-null) and SW480 (mutp53) were selected for further experiments.

As expected, western blot showed that TRIM3 overexpression in HCT116^+/+^ (wtp53) decreased the expression of P21 but had no obvious effect on apoptosis-related proteins such as Bcl-2, Bax, and caspase 3/8. Moreover, in SW480 (mutp53) cells with TRIM3 overexpression, Bcl-2 was down-regulated, and Bax and caspase 3/8 were up-regulated, whereas there was no obvious change on P21. However, there was no significant change on either P21 or apoptosis-related proteins in HCT116^−/−^ (p53-null) cells in the absence of p53 (Fig. 4c).

### The patients of TRIM3 overexpression treated with chemotherapy had better prognosis

Cox univariate analysis suggested that decreased overall survival (OS) and disease-free survival (DFS) were associated with age, pT stage, pN stage, pM stage, and AJCC stage, without TRIM3. Multivariate analysis confirmed that only age and AJCC stage remained independent prognostic factors (Table 3). TRIM3 was not an independent prognostic marker for CRC. Moreover, Kaplan–Meier curves showed there was no significant difference in OS and DFS between TRIM3-positive patients and TRIM3-negative ones (Fig. 4d). Further stratification analysis showed that it induced better outcomes in TRIM3-positive patients treated with chemotherapy than without chemotherapy, especially with FOLFOX and CapeOX. However, there was no significant difference in survival between TRIM3-negative patients with chemotherapy and without chemotherapy (Fig. 4d). Next, we sought to explore the underlying molecular mechanisms of how CRC patients with TRIM3-positive expression could gain benefit from chemotherapy.

**Table 3:** Univariate and multivariate analysis of overall survival and disease‒free survival after surgery (*N* = 348).

	Overall survival				Disease‒free survival			
	Univariate analysis		Multivariate analysis		Univariate analysis		Multivariate analysis	
	HR (95%CI)	*P*	HR (95%CI)	*P*	HR (95%CI)	*P*	HR (95%CI)	*P*
Age								
<50 yr	–	0.006*	–	0.002*	–	0.008*	–	0.004*
50–75 yr	2.204 (0.534–9.102)	0.275	2.282 (0.551–9.448)	0.255	2.109 (0.511–8.710)	0.302	2.152 (0.520-8.913)	0.291
>75 yr	4.082 (0.987–16.882)	0.052	4.474 (1.080–18.530)	0.039*	3.860 (0.933–15.964)	0.062	4.158 (1.004-17.220)	0.049*
Gender								
Male								
Female	0.993 (0.651–1.516)	0.975			0.956 (0.626–1.459)	0.835		
Tumor size								
<4 cm	–	0.352			–	0.405		
4-6 cm	1.331 (0.811–2.185)	0.258			1.308 (0.797–2.147)	0.288		
>6 cm	1.538 (0.826–2.863)	0.175			1.489 (0.800–2.772)	0.209		
Tumor location								
Ascending	–	0.142			–	0.131		
Transverse	0.715 (0.215–2.381)	0.584			0.685 (0.206–2.282)	0.538		
Descending	1.333 (0.634–2.803)	0.448			1.346 (0.641–2.829)	0.433		
Sigmoid	0.533 (0.274–1.035)	0.063			0.532 (0.274–1.034)	0.063		
Rectum	1.098 (0.650–1.854)	0.726			1.104 (0.654–1.864)	0.711		
T stage								
T1	–	0.046*			–	0.043*		
T2	2.154 (0.477–9.723)	0.318			2.123 (0.471–9.582)	0.327		
T3	2.271 (0.551–9.366)	0.256			2.251 (0.546–9.281)	0.262		
T4	3.966 (0.944–16.660)	0.060			3.965 (0.944–16.650)	0.060		
N stage								
N0	–	<0.001*			–	<0.001*		
N1	2.338 (1.458–3.749)	<0.001*			2.294 (1.431–3.677)	<0.001*		
N2	2.769 (1.547–4.958)	0.001*			2.717 (1.518–4.864)	0.001*		
M stage								
M0								
M1	3.426 (1.896–6.192)	<0.001*			3.307 (1.831–5.974)	<0.001*		
AJCC stage								
I	–	<0.001*	–	<0.001*	–	<0.001*	−	<0.001*
II	1.074 (0.501–2.300)	0.855	1.117 (0.521–2.395)	0.775	1.083 (0.505–2.320)	0.838	1.114 (0.520–2.388)	0.781
III	2.706 (1.310–5.588)	0.007*	2.938 (1.420–6.080)	0.004*	2.662 (1.289–5.496)	0.008*	2.874 (1.389–5.947)	0.004*
IV	5.376 (2.294–12.596)	<0.001*	5.632 (2.401–13.211)	<0.001*	5.178 (2.211–12.126)	<0.001*	5.296 (2.259–12.415)	<0.001*
Differentiation								
Well	–	0.186			–	0.232		
Moderate	1.557 (0.966–2.509)	0.069			1.509 (0.937–2.432)	0.091		
Poorly	1.521 (0.580–3.987)	0.394			1.503 (0.573–3.940)	0.408		
TRIM3								
Negative								
Positive	0.911 (0.595–1.394)	0.667			0.904 (0.590–1.384)	0.643		
P53								
Negative								
Positive	1.489 (0.944–2.349)	0.087			1.485 (0.941–2.341)	0.089		

*HR* hazard ratio, *CI* confidence interval.

*P < 0.05 indicated that the 95% CI of HR was not including 1.

We compared the growth curves of RKO (wtp53) and SW480 (mutp53) cells with TRIM3 overexpression treated with 5-Fu and oxaliplatin (regimens of FOLFOX and CapeOX). It was shown that the half-maximal inhibitory concentration (IC50) valve of RKO (wtp53) and SW480 (mutp53) cells with TRIM3 overexpression had no obvious change after treatment with 5-Fu, while IC50 had changed after treatment with oxaliplatin. The IC50 of oxaliplatin in RKO cells (wtp53) with TRIM3 overexpression was increased compared with the control group, but no statistical significance. However, the IC50 of oxaliplatin in SW480 cells (mutp53) with TRIM3 overexpression was significantly decreased (Supplementary Fig. 2a). TRIM3 overexpression could increase the sensitivity of oxaliplatin in mutp53 CRC cells.

### The decreased p53 in the nucleus could alter chemosensitivity to oxaliplatin by affecting MDR1

As shown in the IPA analysis of TRIM3 and p53 downstream pathway (Fig. 4a), TRIM3 could decrease the levels of p53 in the nucleus by retaining p53 in the cytoplasm to alter ABCB1 (ATP-binding cassette subfamily B member 1, MDR1). The decreased wtp53 could arrest cell cycle and activate MDR1. However, the reduced mutp53 in the nucleus could inhibit apoptosis and repress MDR1 to reverse chemotherapy resistance.

To explore the chemosensitivity of CRC cells with TRIM3 overexpression as predicted, we compared the downstream pathway of TRIM3 after treatment with oxaliplatin by western blot. The results showed that the expression of p53 in either RKO (wtp53) or SW480 cells (mutp53) was obviously increased treated with oxaliplatin (Fig. 4e). However, compared with the control, the expression of p53 after TRIM3 overexpression was relatively decreased. Moreover, we analyzed differential expression levels of p53 between the nuclei and cytoplasm. We discovered that the expression of p53 in the cytoplasm was slightly increased, whereas its expression in the nuclei was significantly increased after treatment with oxaliplatin. Nevertheless, the p53 nuclear expression after TRIM3 overexpression was still reduced compared with the control group, which was consistent with the expression of total p53 (Supplementary Fig. 2b). Furthermore, the expression of p21 was reduced, whereas MDR1 protein was increased in RKO cells (wtp53) with TRIM3 overexpression compared with the control group. However, in SW480 cells (mutp53), caspase 3/8 proteins were elevated, while MDR1 expression was decreased in response to TRIM3 overexpression, especially after treatment with oxaliplatin (Fig. 4e).

### Xenograft tumor growth and chemosensitivity of TRIM3 in vivo

**Fig. 5:**
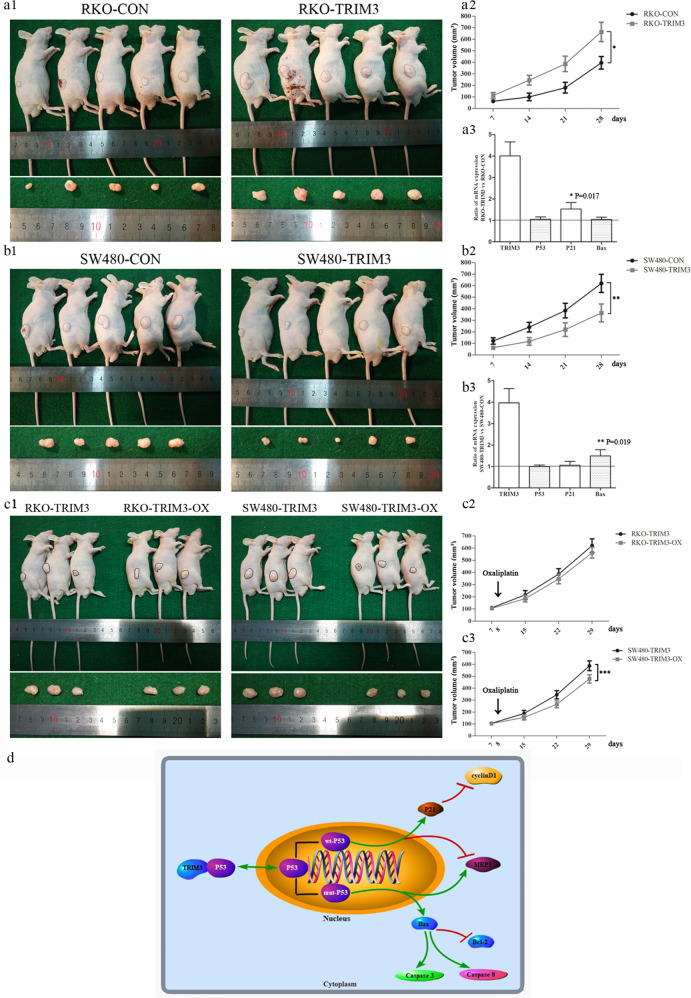
The xenograft tumor growth and chemosensitivity of TRIM3 in vivo and the schematic diagram of TRIM3 dual downstream pathways in CRC. **a1** The xenograft tumor of RKO cells with TRIM3 overexpression in the nude mice; **a2** The tumor volumes of RKO cells for 4 consecutive weeks in the nude mice; **a3** The expression of p53 target genes (P21 and Bax) in the specimen of tumor xenograft of RKO cells by RT-PCR; **b1** The xenograft tumor of SW480 cells with TRIM3 overexpression in the nude mice; **b2** The tumor volumes of SW480 cells in the nude mice for 4 consecutive weeks; **b3** The expression of p53 target genes (P21 and Bax) in the specimen of tumor xenograft of SW480 cells by RT-PCR; **c1** The xenograft tumor of RKO and SW480 cells with TRIM3 overexpression in the nude mice treated with oxaliplatin (5 mg/kg) on the eighth day; **c2** The tumor volumes of RKO cells with TRIM3 overexpression in the nude mice treated with oxaliplatin; **c3** The tumor volumes of SW480 cells with TRIM3 overexpression in the nude mice treated with oxaliplatin; **d** The schematic diagram of TRIM3 dual neoplastic pathways in CRC. All data were expressed as mean ± standard deviation. *, **, ***P < 0.05 in comparison with control groups.

Except in vitro results described above, we proceeded to investigate dual neoplastic features and chemosensitivity of TRIM3 in vivo. As shown in Fig. 5a1/2, the tumor growth index of RKO (wtp53) cells with TRIM3 overexpression was significantly higher than that in the control group. In contrast, the tumor volumes were obviously decreased in the group of SW480 (mutp53) cells with TRIM3 overexpression, compared with the control group (Fig. 5b1/2). Meanwhile, we conducted RT-PCR for the specimen of tumor xenograft to analyze the expression of p53 target genes. The results were consistent with those in vitro. It was shown that p21 mRNA expression was obviously increased, while there was no obvious change of p53 and Bax expression in the tumor xenograft of RKO (wtp53) with TRIM3 overexpression. Meanwhile, Bax mRNA expression was obviously increased, whereas there was no obvious change of p53 and p21 in the tumor xenograft of SW480 (mutp53) with TRIM3 overexpression.

Furthermore, we compared chemosensitivity to oxaliplatin of tumor xenograft in RKO and SW480 cells with TRIM3 overexpression (Fig. 5c1). We discovered that the tumor volumes of RKO (wtp53) cells with TRIM3 overexpression had no significant difference after treatment with oxaliplatin (Fig. 5c2). However, the tumor volumes of SW480 (mutp53) cells with TRIM3 overexpression were obviously decreased after treatment with oxaliplatin (Fig. 5c3).

## Discussion

P53, as a transcription factor, regulates a large number of diverse downstream genes to exert regulative function in multiple signaling processes. It is thought that p53 mutations play a critical role in the CRC adenoma-carcinoma transition process [7].

P53 mutations in CRC not only impair wild-type p53 function (loss-of-function) but also provide neo-morphic (gain-of-function) activities such as promoting cell proliferation, invasion, metastasis, and chemoresistance, thereby promoting cancer progression [5, 8]. Therefore, making mutp53 loss of function or expression inhibition appears a straightforward and logical therapy in CRC [12–14]. Recently, different approaches to target mutant p53 have been exploited including restoration of wild-type-like activities to mutant p53, selective degradation of mutant p53, inhibition of novel protein–protein interactions involved in mediating gain-of-functions of mutant p53, exploitation of synthetic lethality vulnerabilities of mutant p53, inhibition of downstream survival pathways augmented by mutant p53, and immunotherapy based on the recognition of mutant p53 neoantigens [23]. However, current strategies most remain in the theoretical stage, except compound some small molecule inhibitors of p53 [12, 13, 24, 25].

TRIM3 is a member of the TRIM protein superfamily, containing an N-terminal zinc-finger and coiled-coil domains and a C-terminal NHL domain with E3 ubiquitin ligase activity [26]. TRIM3 is identified as the candidate tumor suppressor gene in glioblastomas [21], cervical cancer [27], esophageal squamous cell carcinoma [28], and hepatocellular carcinoma [29]. In human gliomas, TRIM3 is a direct transcriptional target of p53 that, in turn, mediates degradative ubiquitination of the p53 response gene product CDKN1a/p21 [19, 30]. TRIM3 could mediate p53 activity by ubiquitination in a double-edged way. However, the detailed expression and mechanism of TRIM3 in CRC have remained unknown up to date.

It was reported that TRIM3 could function as a tumor suppressor in CRC progression through regulation of p53 protein in CRC cells [31]. However, the expression of TRIM3 from TCGA or Oncomine database displayed discrepant characters between tumor suppressor and promoting tumor, preferred toward tumor suppressor. In our study, the proportion of TRIM3 negative expression was in agreement with the common rate of p53 mutation in CRC. Moreover, it was interesting to find that TRIM3 demonstrated dual neoplastic features related to p53 status (wild-type/mutant) in cell experiments. TRIM3 could act as a pro-tumorigenic gene in wtp53 cells (LoVo, RKO), while TRIM3 could serve as a tumor-suppressive gene in mutp53 cells (SW480, HT29). Moreover, TRIM3 was discovered to directly interact with p53 at the C terminus of p53 (residues 320 to 393), the common segment of wtp53 and mutp53. TRIM3 overexpression in either wtp53 or mutp53 cells could retain p53 in the cytoplasm and decrease its expression in the nucleus, but made no change in total protein levels. Furthermore, TRIM3 exerted dual roles on neoplastic features in a p53-dependent manner confirmed by using three p53-specific cell lines. TRIM3 could decrease the expression level of p53 in the nucleus to influence cell cycle or apoptosis by retaining p53 in the cytoplasm dependent on p53 wild or mutant status (Fig. 5d). The decreased wtp53 could arrest the cell cycle to exert promoting cancer, while the reduced mutp53 could inhibit apoptosis to play a role of suppressing cancer (Fig. 5d). That was the reason why TRIM3 demonstrated dual neoplastic features of pro-tumorigenic gene in wtp53 cells and tumor-suppressive gene in mutp53 cells.

Generally, p53 mutant proteins get gain-of-function (GOF) properties via protein-protein interactions with other transcription factors and co-factors, or by binding directly to novel target genes [23]. However, wild-type p53 mediates cellular responses by transactivating MDM2 for proteasomal degradation and ubiquitination in a negative feedback loop. Our team discovered TRIM3 mediated p53 by direct interaction with p53 at the C terminus of p53 (residues 320 to 393) in CRC. Therefore, our strategy that TRIM3 made the nuclear expression of mutp53 suppression by retaining mutp53 in the cytoplasm belonged to a kind of selective degradation of mutant p53.

Chemotherapy is the basis of CRC treatment, except for surgery and radiotherapy [32]. However, chemotherapy therapy provides only a limited increase in overall survival due to chemotherapy resistance after treatment, especially for these advanced CRC patients. Moreover, chemotherapy resistance in CRC was often associated with overexpression of MDR1 gene, the most frequently expressed drug resistance gene in human tumors [33]. MDR1 overexpression has rendered many currently available chemotherapeutic agents ineffective. Studies have shown that inhibition of MDR1 could reverse drug resistance in CRC [34]. Our results showed the patients with TRIM3 overexpression treated with chemotherapy had a better prognosis than those without chemotherapy. The chemosensitivity of oxaliplatin was significantly increased in mutp53 CRC cells after TRIM3 overexpression by decreasing wtp53 in the nucleus to inactivate MDR1. In actuality, there was substantial evidence that p53 was involved in the regulation of MDR1. The mutp53 specifically stimulated MDR1 promoter activity, whereas wtp53 exerted specific repression [35]. Thus, making mutp53 loss function or inhibition expression could inhibit MDR1 and reverse the chemotherapy resistance of oxaliplatin [36].

In a word, our study was the first to demonstrate that TRIM3 could display dual neoplastic features of a pro-tumorigenic gene in wtp53 cells and a tumor-suppressive gene in mutp53 cells. TRIM3 could exert dual roles in colorectal carcinogenesis and alter chemosensitivity by retaining p53 in the cytoplasm to decrease its nuclei expression through direct binding with each other. Moreover, TRIM3 could successfully reverse the chemotherapy resistance of oxaliplatin in mutp53 cells by downregulation MDR1. Therefore, TRIM3 was a potential therapeutic strategy to improve the survival of CRC patients with mutp53 by degradation of mutp53 in the nuclei.

## Materials and methods

### Patients and tissue specimens

A total of 32 couples of fresh tissue specimens (including tumor tissues and adjacent non-cancerous mucosa) were collected from CRC patients who underwent radical surgery at Shanghai General Hospital and Fudan University Shanghai Cancer Center and for qRT-PCR and western blot. Moreover, a total of 348 pairs of preserved paraffin-embedded human CRC tissues and adjacent non-cancerous mucosa specimens were retrieved from the tumor tissue bank. The group comprised 169 males and 179 females. The number of patients with stage I, II, III, and IV was 60, 155, 109, and 24 cases, respectively. These 348 pair samples were constructed into a tissue microarray to simultaneously detect protein expression in various tissues [37]. None of the patients underwent preoperative chemotherapy or radiation therapy. 75.9% (264/348) of the patients received postoperative adjuvant chemotherapy. The detailed adjuvant chemotherapy characteristics of these 264 patients were presented in Supplementary Table 1. The four common chemotherapy regimens in our cohort were 5-Fu/LV (12 patients), Capecitabine (29 patients), FOLFOX (141 patients) and CapeOX (76 patients). Pathological diagnoses were confirmed by two pathologists, according to the American Joint Committee on Cancer (AJCC).

### Cell culture and transfection

Human CRC cell lines (LoVo, HT29, RKO, SW480, and HCT116) were purchased from Shanghai Institute Cell Bank, Chinese Academy of Science (original source: American Type Culture Collection, ATCC, Manassas, VA, USA). HCT116^p53−/−^ was kindly provided by Professor Chuan-gui Wang (Translational Medicine Research Institute, Shanghai General Hospital, China). Cells were cultured in DMEM supplemented with 10% fetal bovine serum (FBS), 100 U/ml penicillin and 100 mg/ml streptomycin, and maintained at 37 °C and 5% CO2 in a humidified incubator.

To establish cell lines with stable knockdown and overexpression, TRIM3 shRNA and overexpression lentiviral vectors were commercially designed and constructed by Shanghai Genechem Company (Shanghai, China).

### RNA extraction, reverse transcription and qRT-PCR

RNA extraction and reverse transcription methods were performed as described previously [38]. The primers used in this study were listed in Supplementary Table 2A.

### Western blot

Total proteins were extracted and western blot was performed as described previously [38]. Proteins (40 mg) were separated for western blot analysis. The detailed antibodies and their concentration were described in Supplementary Table 2B.

### Immunohistochemistry

Immunohistochemistry was performed as previously described [38]. The detailed antibodies and their concentration were described in Supplementary Table 2B. The evaluation was based on the staining intensity and extension as described elsewhere [38].

### Ingenuity pathway analysis

To predict the relationship between TRIM3 and p53 and their downstream pathway, we performed pathway in-silico analysis with Ingenuity Pathway Analysis (IPA) software (Ingenuity Inc., CA, USA). TRIM3 and p53 were used as seeds to construct their related interaction network available in the Ingenuity database.

### Cell counting Kit-8 assay

The proliferation of CRC cells was assessed using a CCK-8 assay (DOJINDO, Kumamoto, Japan), in accordance to manufacturer’ instruction. The detailed concentrations of the four types of chemotherapy in our study were described in Supplementary Table 2C.

### Colony formation assay

The colony formation assay was performed as previously described [38].

### Migration and invasion assay

The assays were performed using standard techniques as described previously [38].

### Flow cytometry analysis of the cell apoptosis and cycle

Cell apoptosis was analyzed using the Annexin V-PE /7AAD Apoptosis Detection kit (BD, CA, USA). The cells were collected and resuspended in 500 μl of 1 × binding buffer. The cells were then stained for 15 min in the dark at room temperature using the apoptosis kit in accordance with the manufacturer’s instructions. The apoptotic cells were analyzed by flow cytometry (BD Accuri C6, USA). For the cell cycle, cells were harvested and fixed with 5 mL of prechilled 70% ethanol overnight at 4 °C. The cells were then gently centrifuged and stained for 15 min by the Cell Cycle kit (BD, CA, USA). The cells were analyzed by flow cytometry (BD Accuri C6, USA).

### Immunofluorescence

Cells plated on Nunc Glass Bottom Dish (ThermoFisher Scientific, MA, USA) were allowed to grow for 48 h, fixed with 4% paraformaldehyde (Sangon Biotech, Shanghai, China), permeabilized with 0.1% Triton X-100 (Sangon Biotech, Shanghai, China). Dishes were incubated overnight at 4 °C with anti-TRIM3 rabbit monoclonalantibody at 1:200 (abcam, CA, USA) and anti-p53 mouse monoclonal antibody at 1:250 (CST, CA, USA). Secondary antibody staining was performed with anti-rabbit and anti-mouse (CST, CA, USA), mounted with DAPI (CST, CA, USA), and imaged with confocal microscope.

### Co-immunoprecipitation

293 T cells with overexpression TRIM3 or/and p53 were lysed in ice cold HEPES buffer (50 mM HEPES, pH 7.5, 150 mM NaCl, 1 mM MgCl2, 1 mM EGTA) containing protease inhibitors. After complete lysis by sonication, the lysates were centrifuged at 14,000 rpm to collect the supernatants. The extracts were incubated with antibodies against Flag and HA overnight at 4 °C, followed by the addition of Protein A plus-Sepharose and incubation for 4 h at 4 °C. After five times wash with cell-lysis buffer, the beads were boiled with SDS loading buffer and then analyzed by SDS-PAGE and western blotting. Moreover, to analyze the interaction between TRIM3 and p53, we constructed HA-tagged wtp53 and C-terminal(320-393) truncation mutp53 [39]. The subsequent CoIP followed the same steps mentioned above.

### Nude mice xenograft models

To clarify the effect of TRIM3 in vivo, four-week-old male BALB/C nude mice purchased from the Institute of Zoology, at the Chinese Academy of Sciences of Shanghai were used to establish CRC xenografts. CRC cells (5 × 10^6^) with TRIM3 overexpression were suspended in 100 μL of PBS and subcutaneously injected into flanks of the mice. To verify the chemosensitivity to oxaliplatin of TRIM3 overexpression in vivo, a single dose of 5 mg/kg oxaliplatin was injected via tail intravenous after 8 days of establishment xenograft models of TRIM3 overexpression. Tumor volume (mm^3^) was estimated weekly by using the following formula: tumor volume (mm^3^) = length × width^2^ × 0.5 [40]. All the mice were sacrificed after 4 weeks. All animal studies were performed in accordance with the animal care guidelines. All efforts were made to minimize animal suffering.

### Statistical analysis

All data and survival analyses were calculated by using SPSS version 19.0 statistical software (SPSS, Chicago, USA). All experiments were conducted in triplicate to verify the reproducibility of the findings. The significance of the covariate differences was determined using a two-tailed χ2 or Fisher’s exact tests where appropriate. The survival rates were calculated by the Kaplan–Meier method and the differences between the survival curves were examined by the log-rank test. A Cox proportional hazards model was used to investigate the multivariate analysis and independent prognostic factors. The in vitro and in vivo data were expressed as the mean ± SD and were determined by the two-tailed Student’s *t*-test. All *P* < 0.05 were considered to be statistically significant.

## Supplementary information

Figure S1

Figure S2

Supplementary Table 1

Supplementary Table 2

Figure 1-Original Data

Figure 2-Original Data

Figure 3-Original Data

Figure 4-Original Data

Figure 5-Original Data

## Data Availability

The data and material that were used or analyzed during the current study are available from the corresponding author on reasonable request.
