# Supplementary figures and images for: Dual roles of TRIM3 in colorectal cancer by retaining p53 in the cytoplasm to decrease its nuclear expression
Source: Cell Death Discov. 2023 Mar 9;9:85. doi: 10.1038/s41420-023-01386-1 (PMC9998637; doi:10.1038/s41420-023-01386-1)

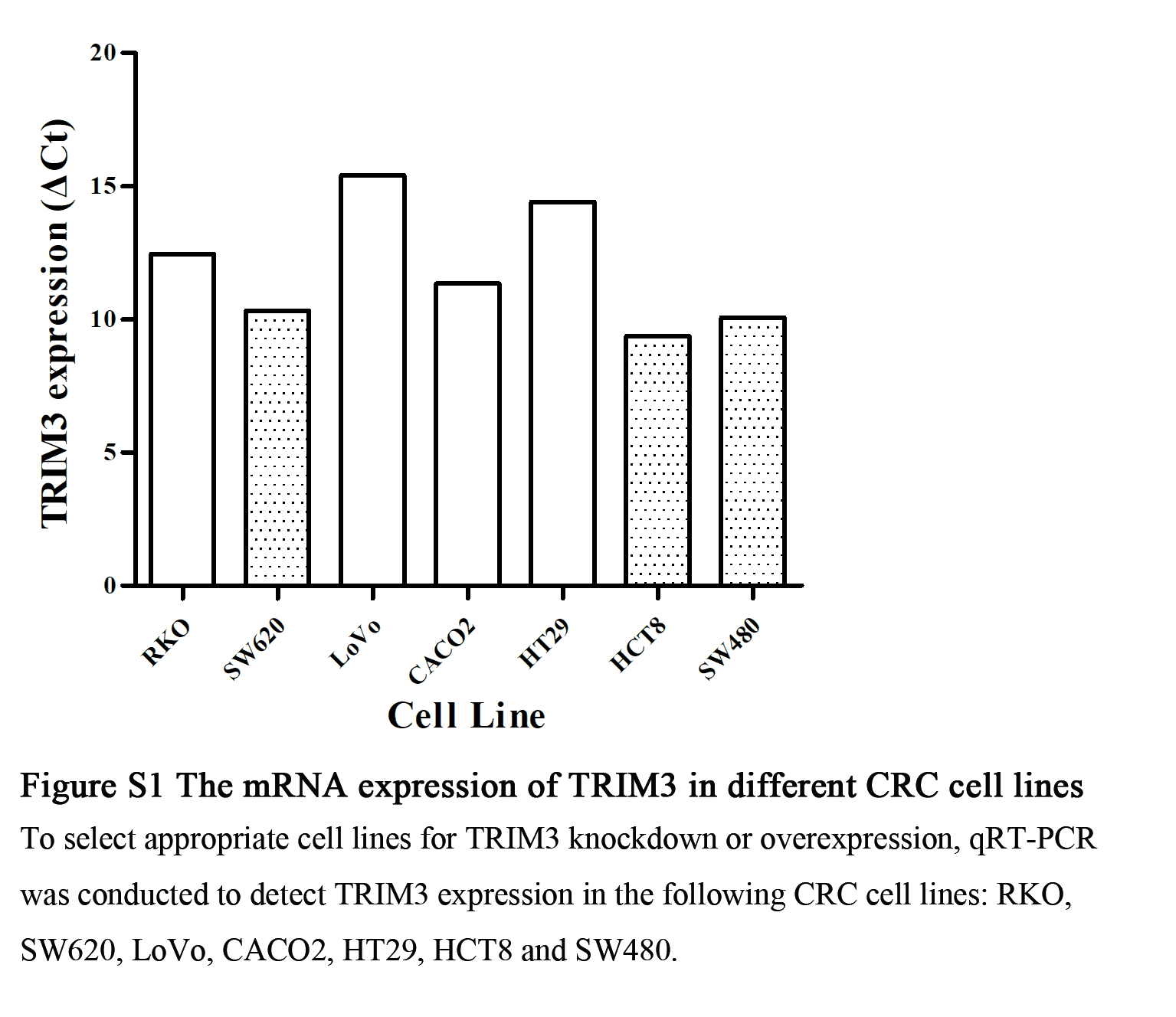

Supplement: Supplementary file 1 — Figure S1 [file 41420_2023_1386_MOESM1_ESM.tif]

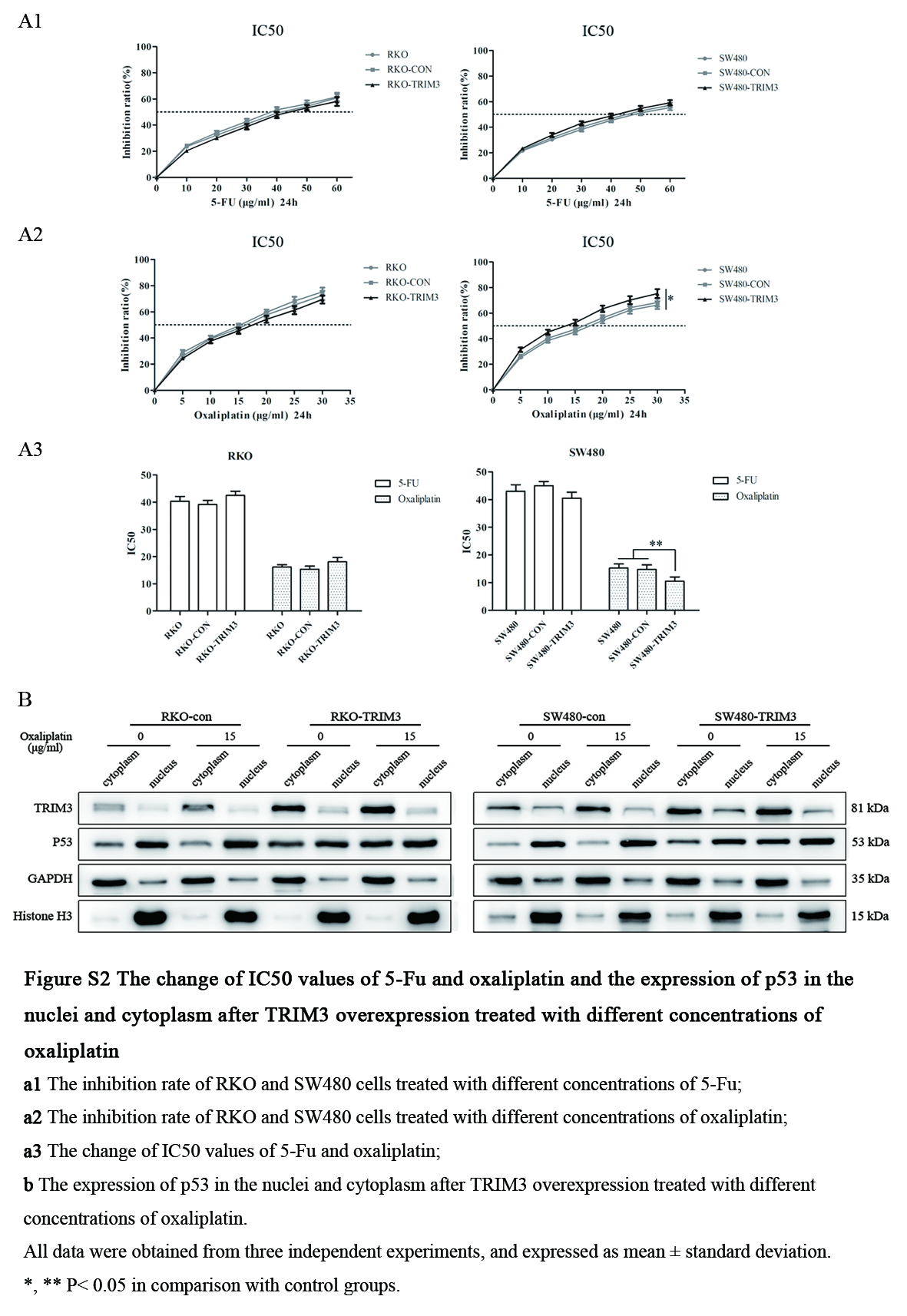

Supplement: Supplementary file 2 — Figure S2 [file 41420_2023_1386_MOESM2_ESM.tif]
