# Supplementary material for: Dual roles of TRIM3 in colorectal cancer by retaining p53 in the cytoplasm to decrease its nuclear expression
Source: Cell Death Discov. 2023 Mar 9;9:85. doi: 10.1038/s41420-023-01386-1 (PMC9998637; doi:10.1038/s41420-023-01386-1)
Supplement: Supplementary file 3 — Supplementary Table 1 [file 41420_2023_1386_MOESM3_ESM.docx]

|  | Adjuvant Chemotherapy | N |
| --- | --- | --- |
| No | None | 84 |
| Single-agent | 5-Fu/LV | 12 |
|  | Oxaliplatin | 1 |
|  | Capecitabine | 29 |
|  | Irinotecan hydrochloride | 1 |
| Combination therapies | 5-Fu/LV+ Oxaliplatin | 141 |
|  | 5-Fu/LV+Irinotecan hydrochloride | 1 |
|  | Capecitabine+ Cisplatin | 2 |
|  | Capecitabine+ Oxaliplatin | 76 |
|  | Capecitabine+5-Fu/LV | 1 |

Supplementary Table 1. Adjuvant chemotherapy for CRC patients in our cohort (N=348)
