# Supplementary material for: Dual roles of TRIM3 in colorectal cancer by retaining p53 in the cytoplasm to decrease its nuclear expression
Source: Cell Death Discov. 2023 Mar 9;9:85. doi: 10.1038/s41420-023-01386-1 (PMC9998637; doi:10.1038/s41420-023-01386-1)
Supplement: Supplementary file 4 — Supplementary Table 2 [file 41420_2023_1386_MOESM4_ESM.doc]

**Supplementary Table 2A. Primer sequences used for qPCR in this study**

| Gene name (Human) | Primer sequence (5′-3′) | |
| --- | --- | --- |
|  | Forward | Reverse |
| TRIM3 | GCGACCTGGAGACCATTTGT | GCTACTGCCGATGTGTTCCTG |
| GAPDH | GGAGCGAGATCCCTCCAAAAT | GGCTGTTGTCATACTTCTCATGG |
| TP53 | CAGCACATGACGGAGGTTGT | TCATCCAAATACTCCACACGC |
| CDKN1A | TGTCCGTCAGAACCCATGC | AAAGTCGAAGTTCCATCGCTC |
| BAX | CCCGAGAGGTCTTTTTCCGAG | CCAGCCCATGATGGTTCTGAT |

**Supplementary Table 2B. Antibodies applied in this study**

| Antibody | IHC | WB | IP | Company | Art.No |
| --- | --- | --- | --- | --- | --- |
| TRIM3 | 1:200 | 1:500 | - | Abcam | ab111840 |
| P53 | 1:250 | 1:1000 | - | CST | 48818 |
| P21 | - | 1:1000 | - | Abcam | ab109520 |
| MDR1 | - | 1:1000 | - | Abcam | ab170904 |
| Bax | - | 1:800 | - | Abcam | ab32503 |
| Bcl-2 | - | 1:800 | - | Abcam | ab32124 |
| Caspase 3 |  | 1:1000 |  | Abcam | ab32351 |
| Caspase 8 |  | 1:1000 |  | Abcam | ab32125 |
| Flag | - | - | 1:50 | Abcam | ab49763 |
| HA | - | - | 1:50 | Abcam | ab9110 |
| v5 |  |  | 1:50 | Abcam | ab27671 |
| Histone H3 |  | 1:1000 |  | Abcam | ab1791 |

| Chemotherapy | Concentrations | Company |
| --- | --- | --- |
| 5-Fu^1^ | 25μM | Selleck |
| Oxaliplatin^2^ Capecitabine^3^ Epirubicin^4^ | 100μM | Selleck |
|  | 1 mM | Selleck |
|  | 20μM | Selleck |

**Supplementary Table 2C. The Concentrations of four kinds of chemotherapy in our study**

Reference:

1.http://www.selleckchem.com/products/Adrucil(Fluorouracil).html

2.http://www.selleckchem.com/products/Eloxatin.html

3.http://www.selleckchem.com/products/Capecitabine(Xeloda).html

4.http://www.selleckchem.com/products/Ellence.html
