# Supplementary material for: Dual roles of TRIM3 in colorectal cancer by retaining p53 in the cytoplasm to decrease its nuclear expression
Source: Cell Death Discov. 2023 Mar 9;9:85. doi: 10.1038/s41420-023-01386-1 (PMC9998637; doi:10.1038/s41420-023-01386-1)
Supplement: Supplementary file 5 — Figure 1-Original Data [file 41420_2023_1386_MOESM5_ESM.pdf]

Figure 1A

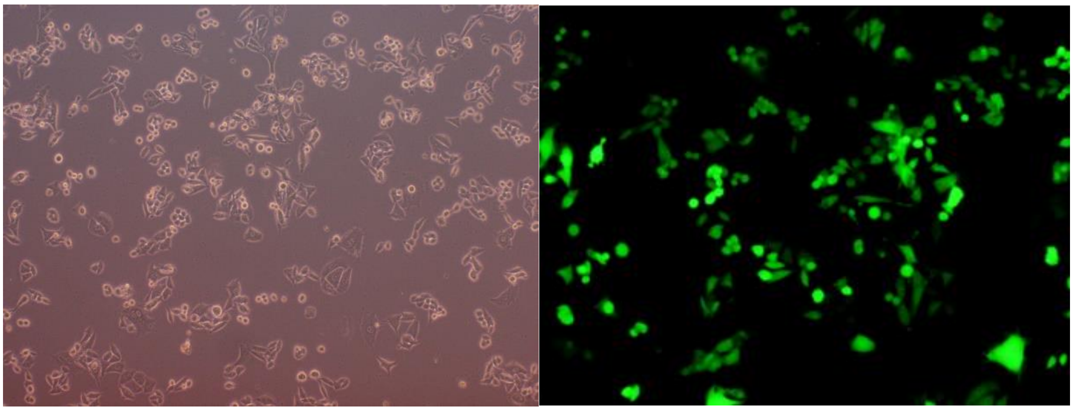

RKO-CON-24H

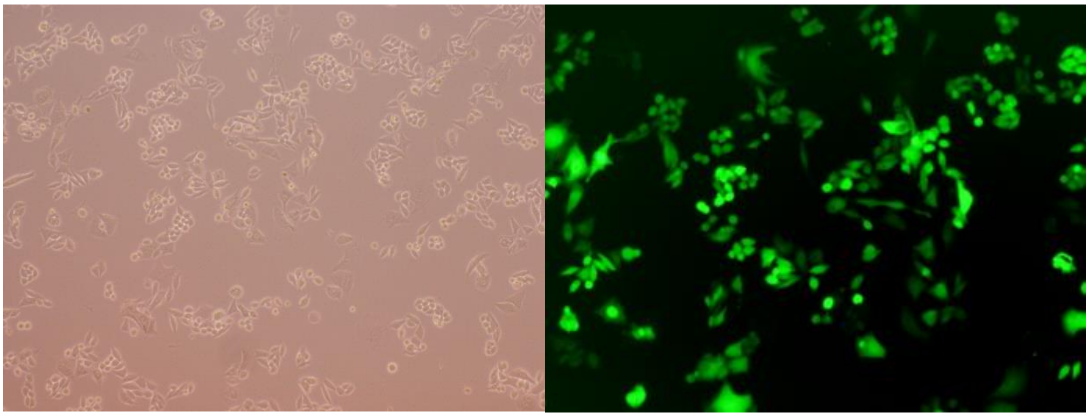

RKO-CON-36H

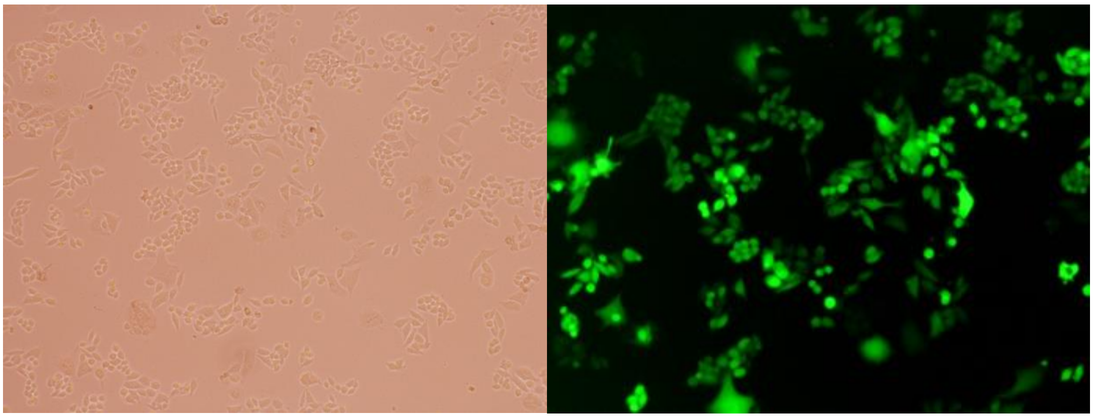

RKO-CON-48H

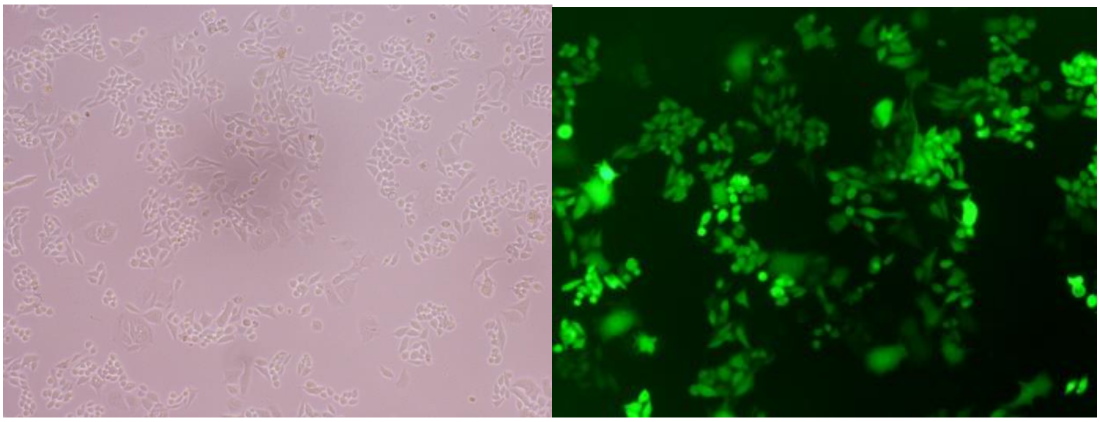

RKO-CON-60H

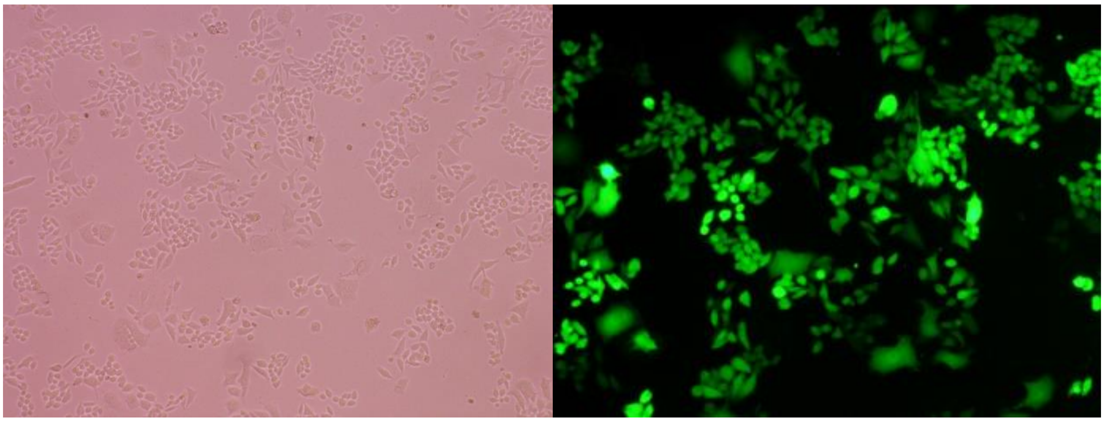

RKO-CON-72H

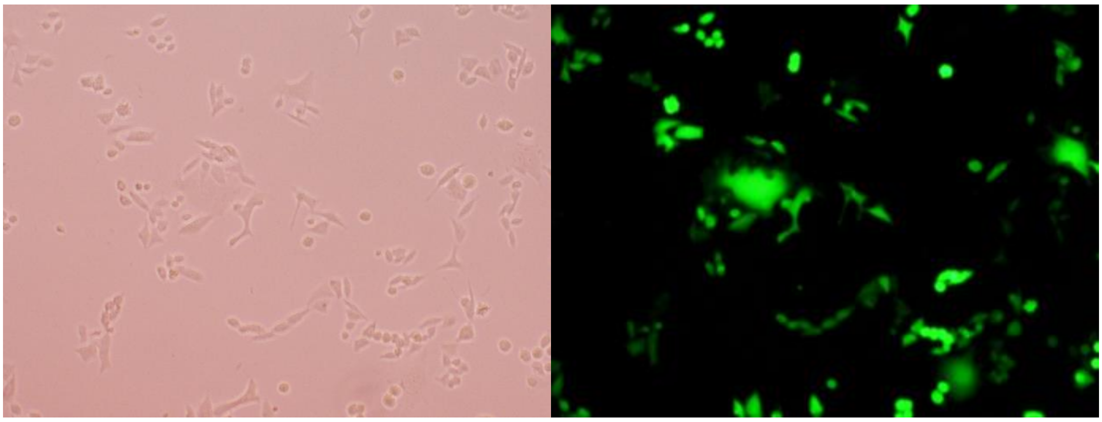

RKO-shTRIM3-24H

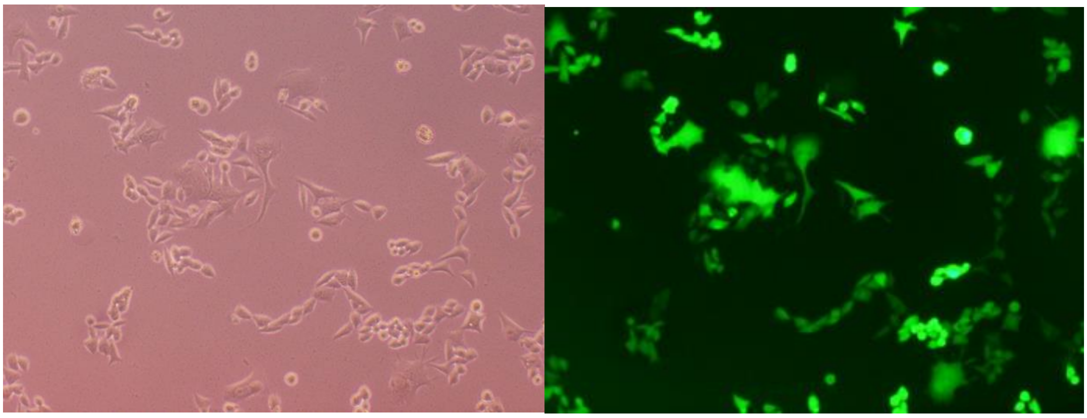

RKO-shTRIM3-36H

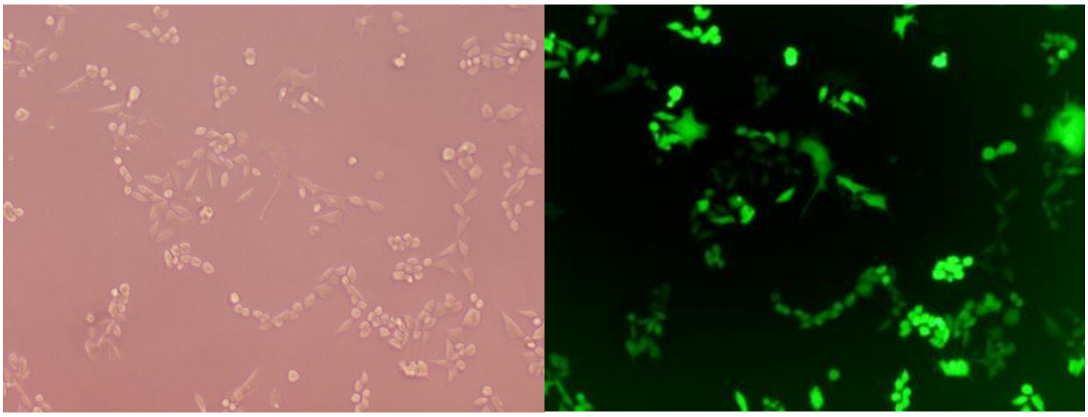

RKO-shTRIM3-48H

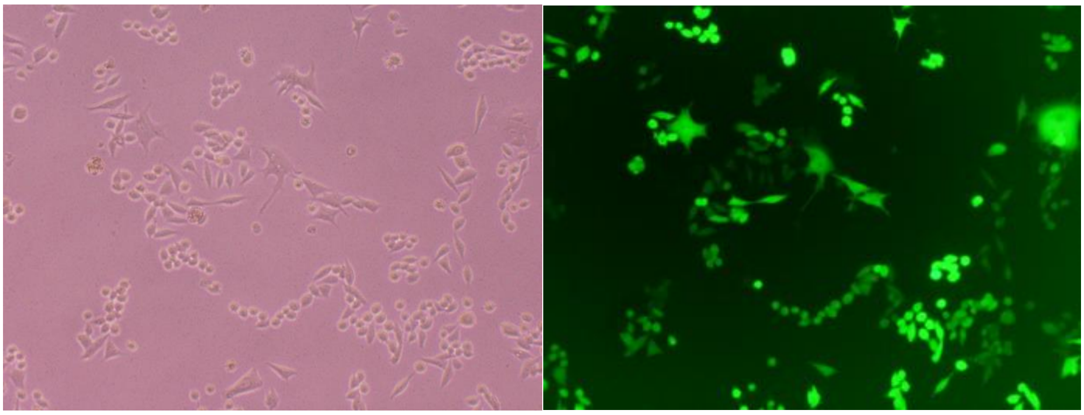

RKO-shTRIM3-60H

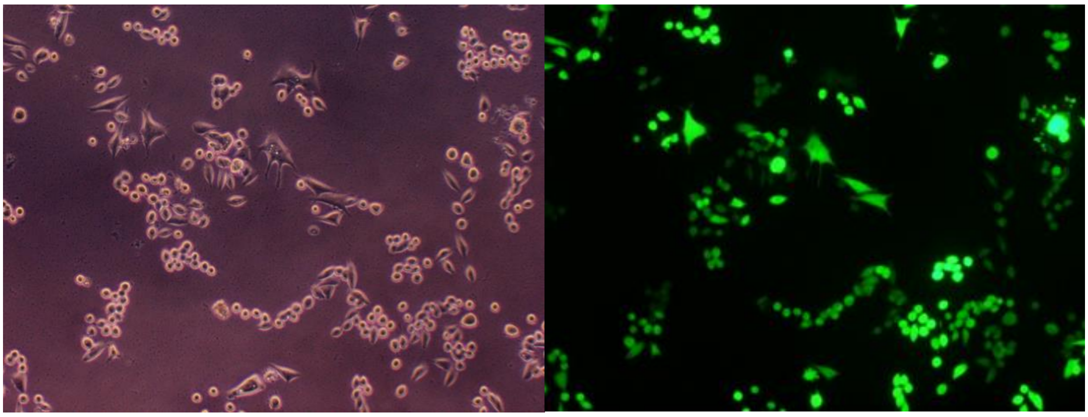

RKO-shTRIM3-72H

Figure 1C

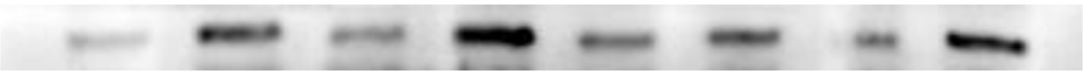

TRIM3-1

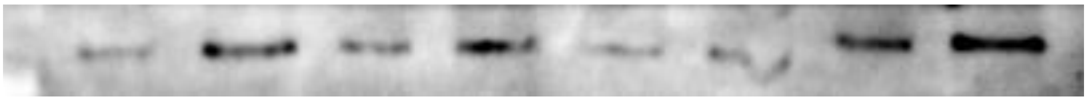

TRIM3-2

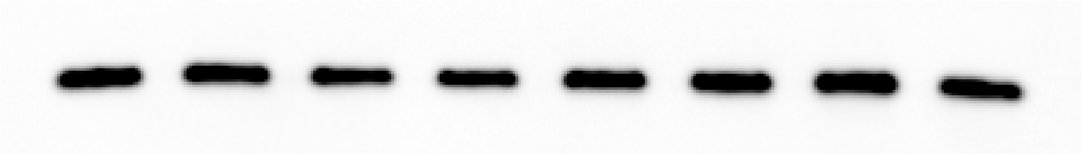

GAPDH-1

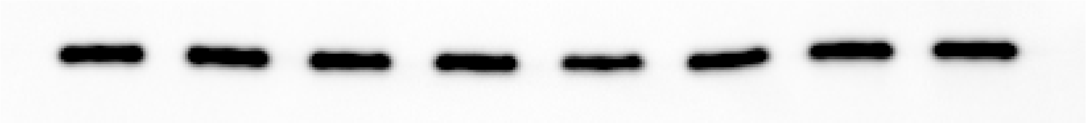

GAPDH-2

Figure 1D

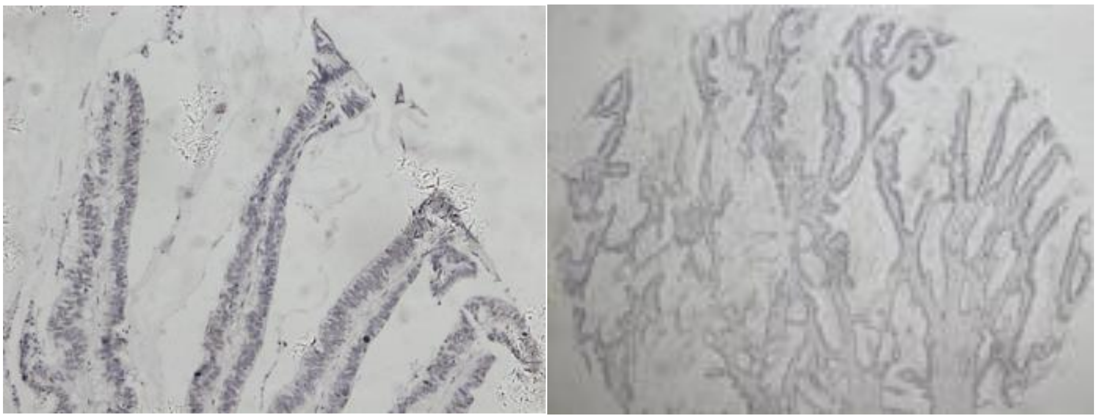

Normal-1

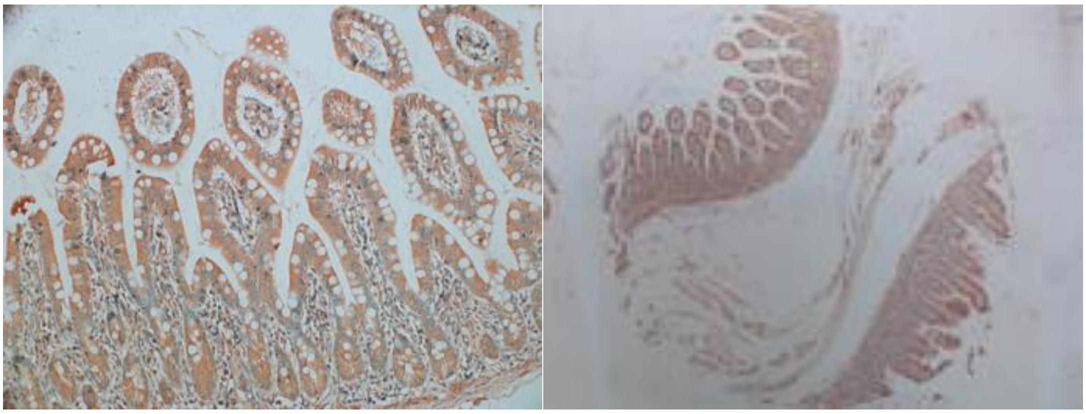

Normal-2

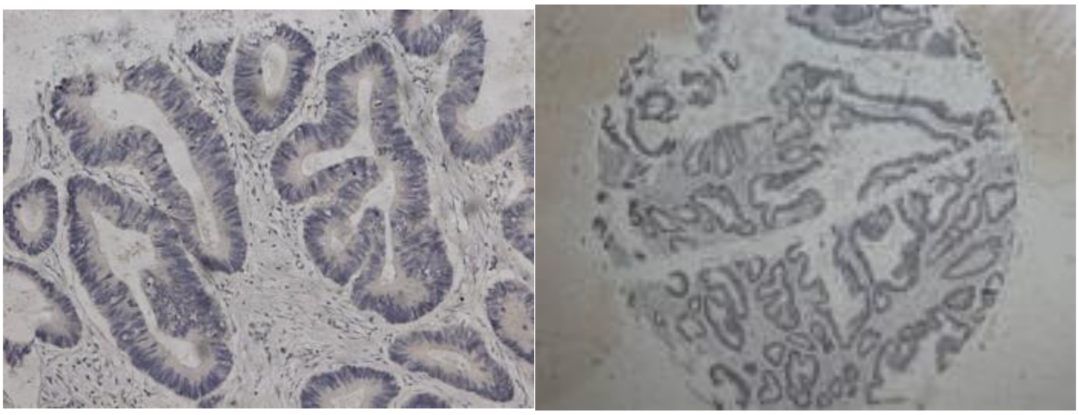

Well-differentiated-1

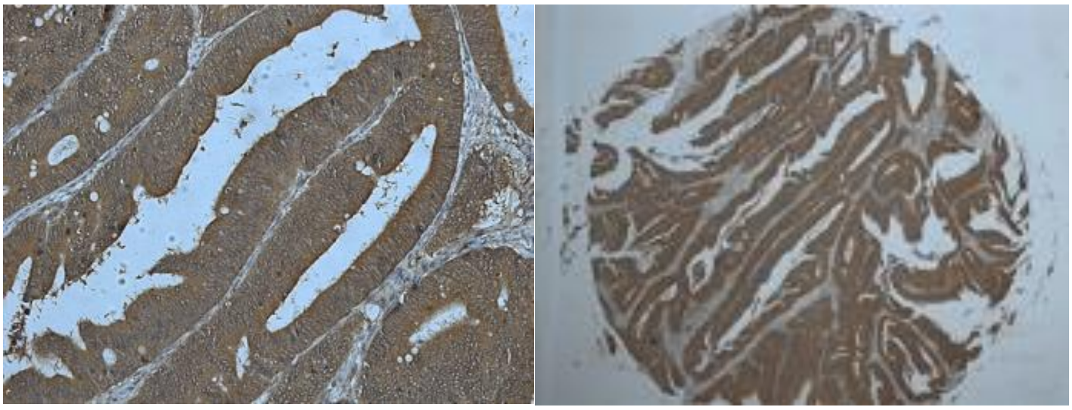

Well-differentiated-2

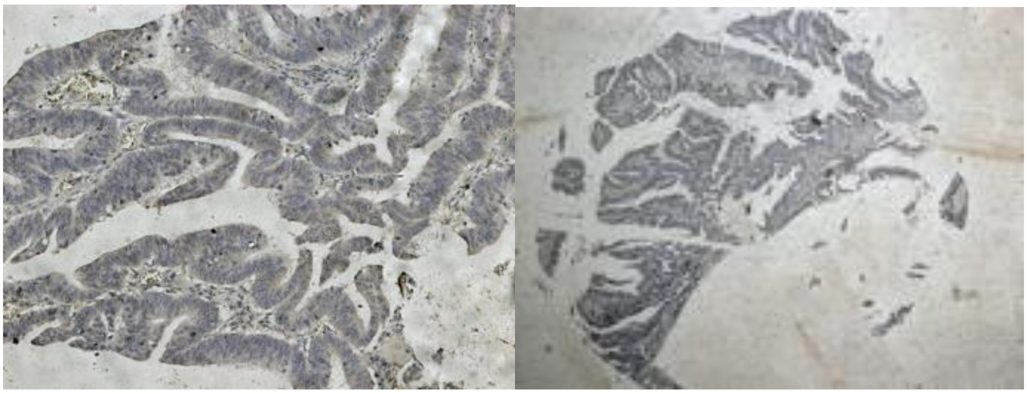

Moderately-differentiated-1

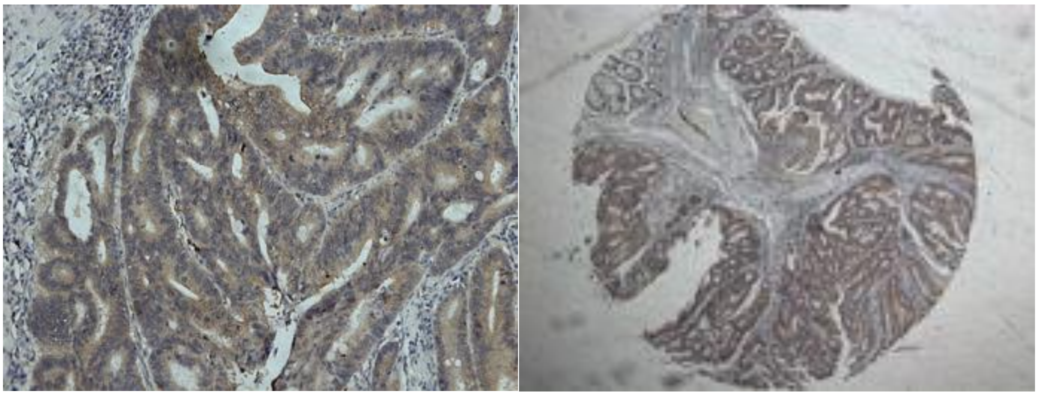

Moderately-differentiated-2

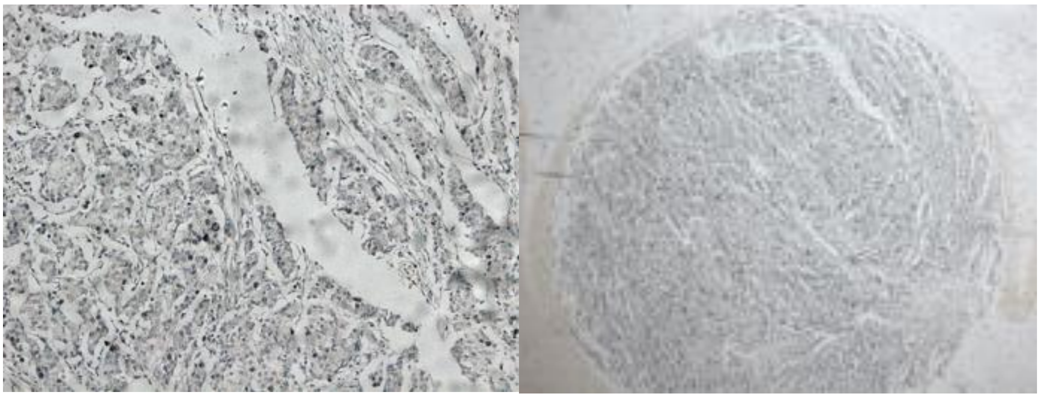

Poor-differentiated-1

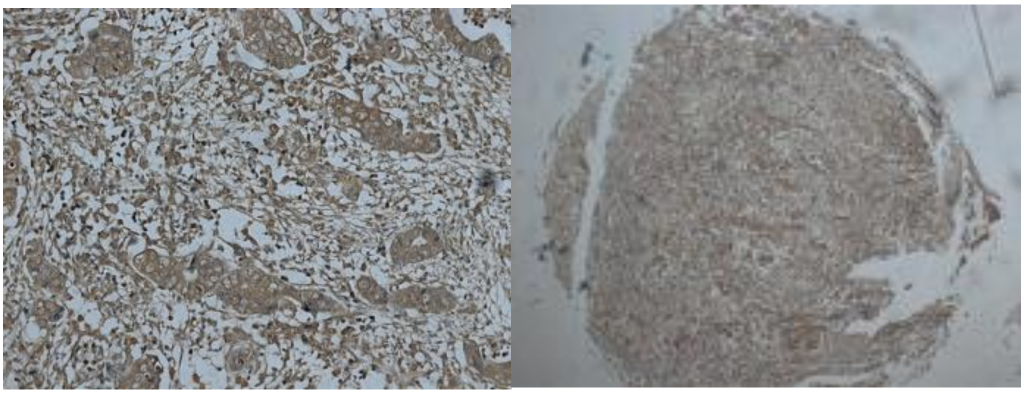

Poor-differentiated-2
