# Supplementary material for: Dual roles of TRIM3 in colorectal cancer by retaining p53 in the cytoplasm to decrease its nuclear expression
Source: Cell Death Discov. 2023 Mar 9;9:85. doi: 10.1038/s41420-023-01386-1 (PMC9998637; doi:10.1038/s41420-023-01386-1)
Supplement: Supplementary file 6 — Figure 2-Original Data [file 41420_2023_1386_MOESM6_ESM.pdf]

# Figure 2B1

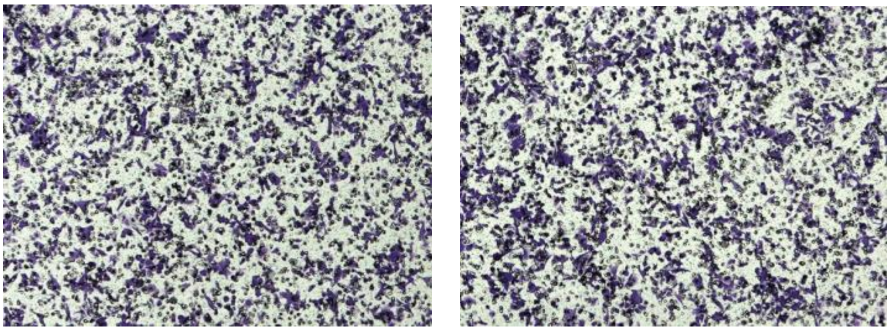

Migration-LOVO

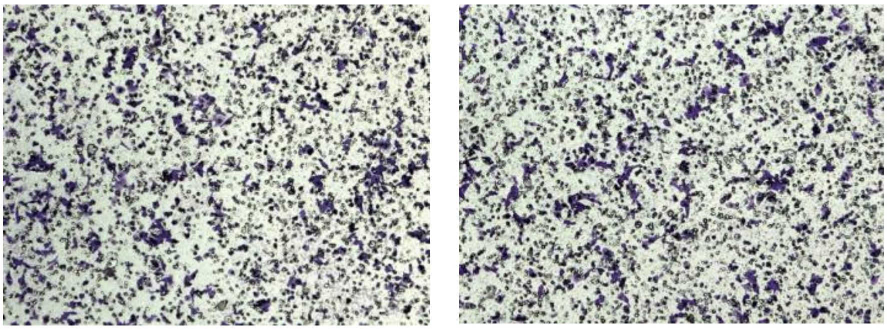

Migration-LOVO-shTRIM3

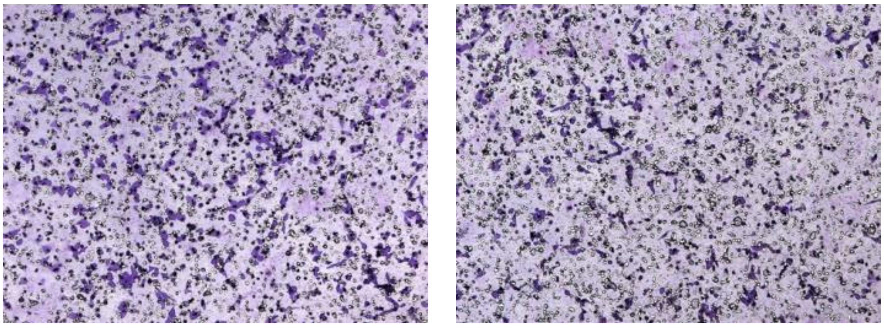

Invasion-LOVO

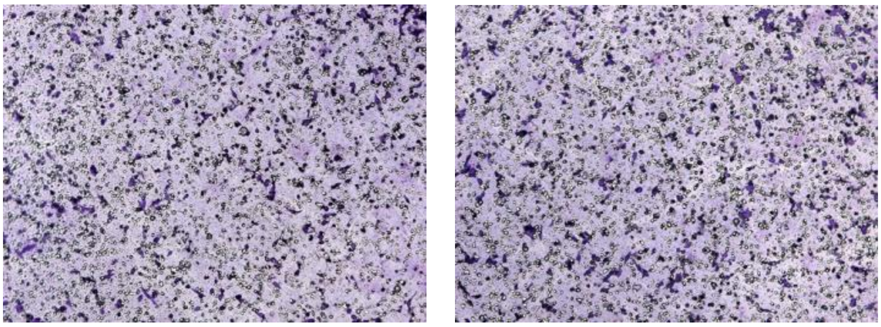

Invasion-LOVO-shTRIM3

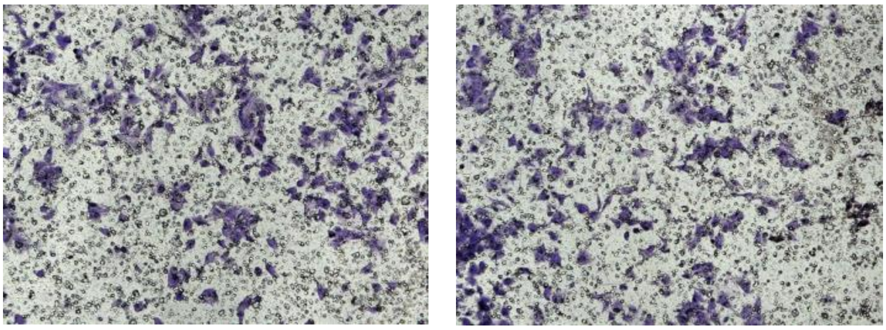

Migration-HT29

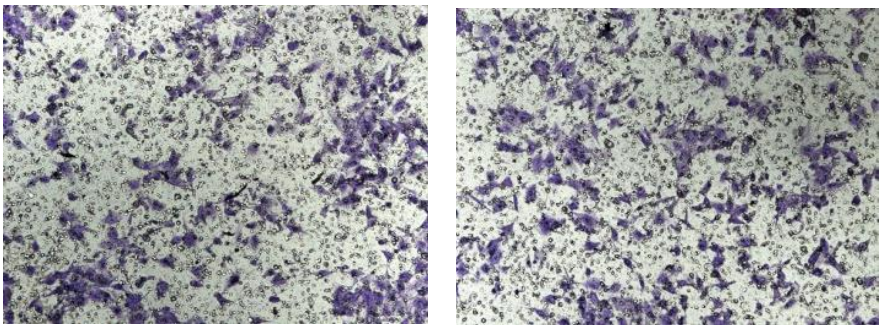

Migration-HT29-shTRIM3

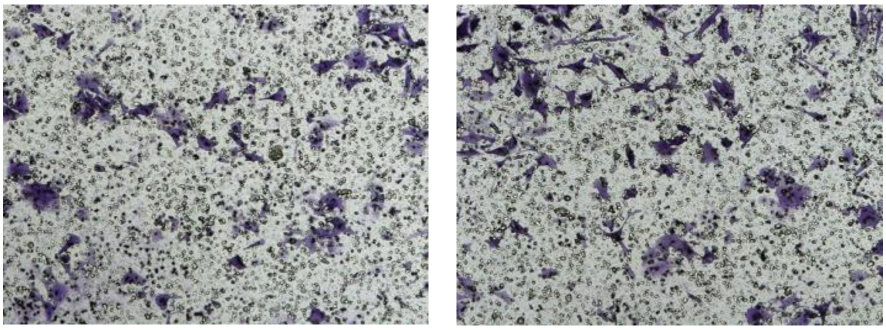

Invasion-HT29

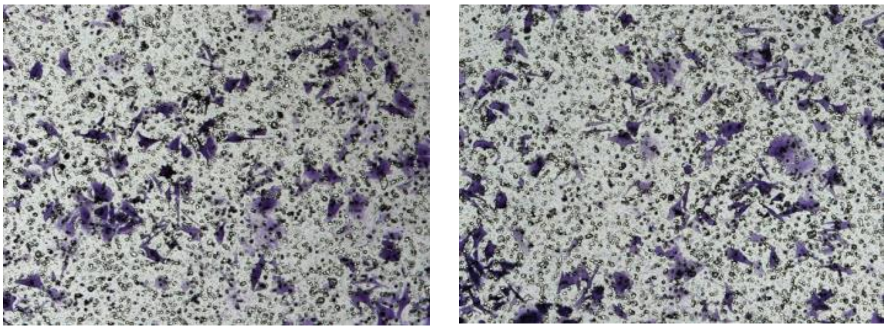

Invasion-HT29-shTRIM3

Figure 2B2

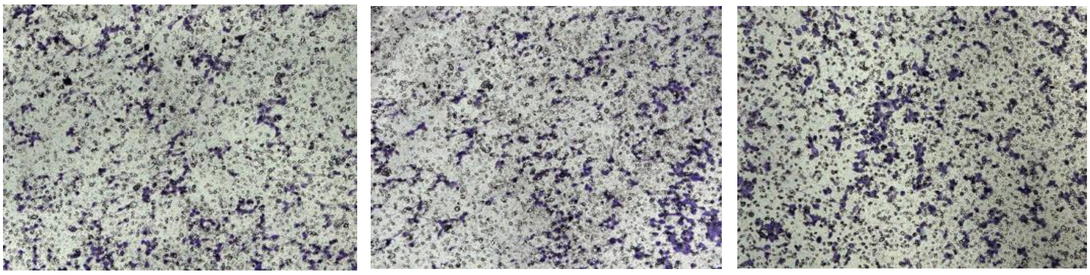

Migration-RKO

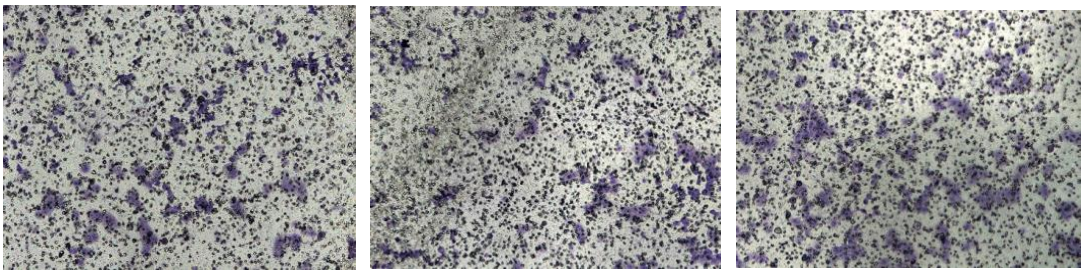

Invasion-RKO

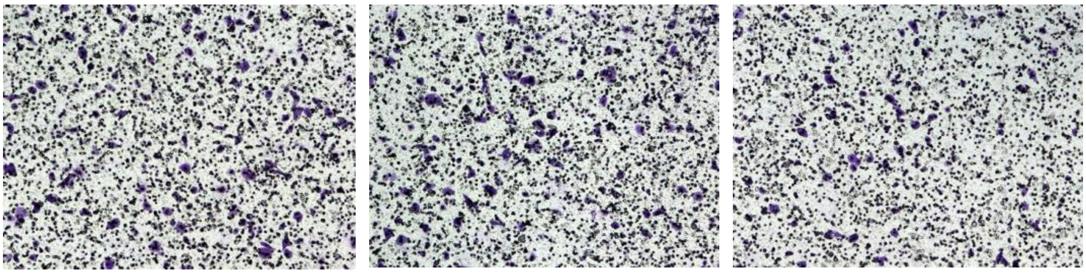

Migration-SW480

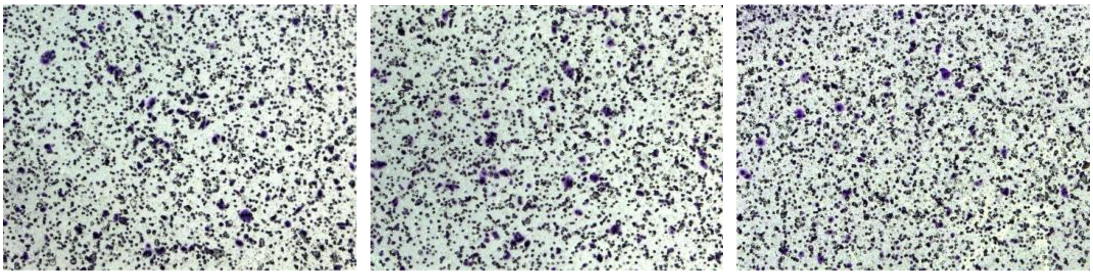

Invasion-SW480

# Figure 2C1

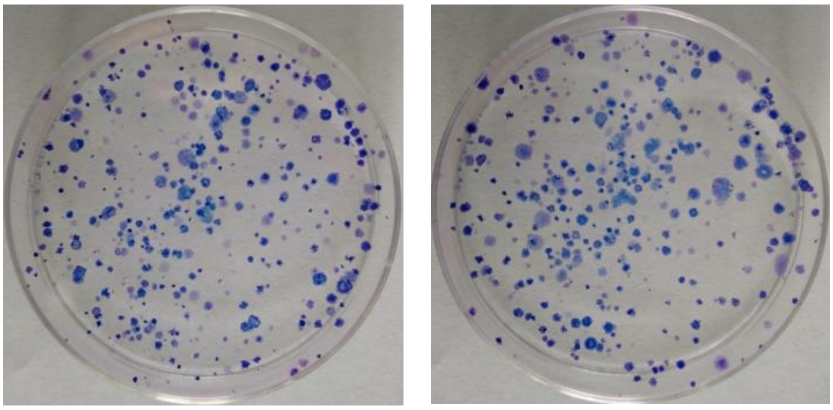

LOVO

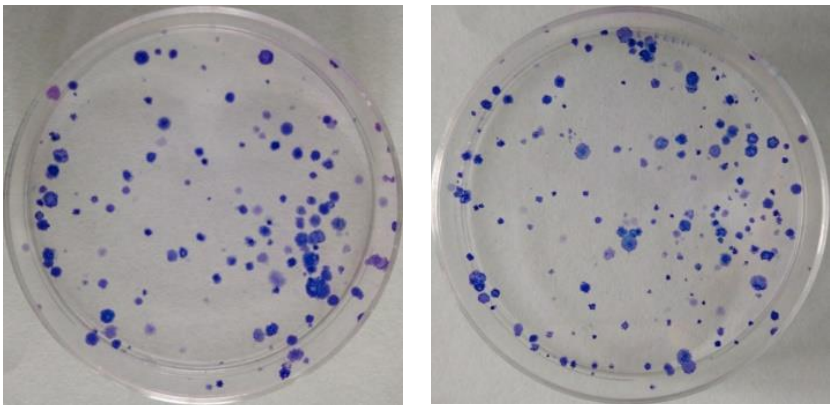

LOVO-shTRIM3

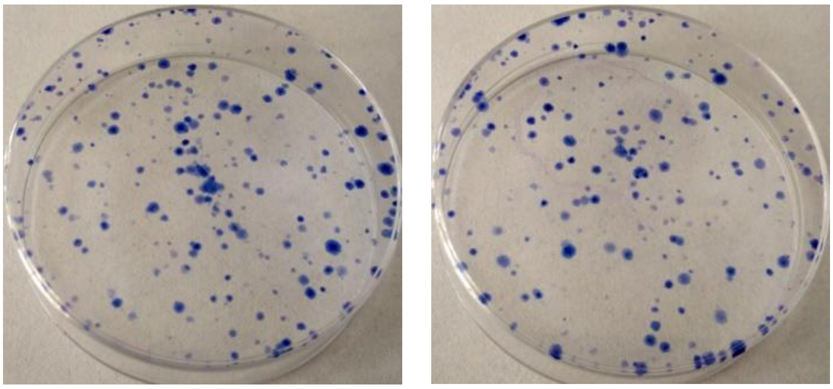

HT29

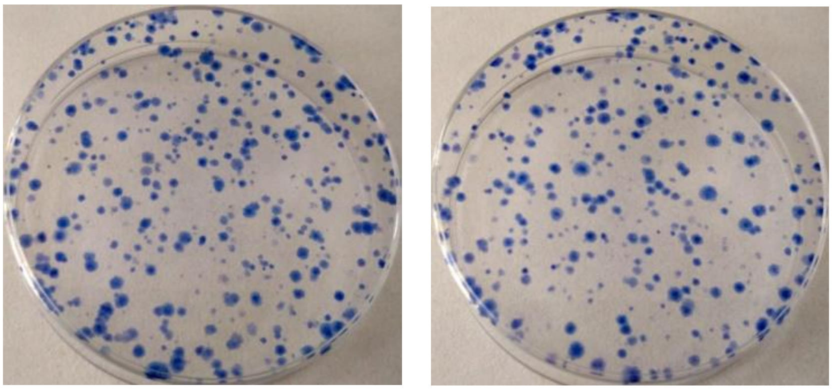

HT29-shTRIM3

# Figure 2C2

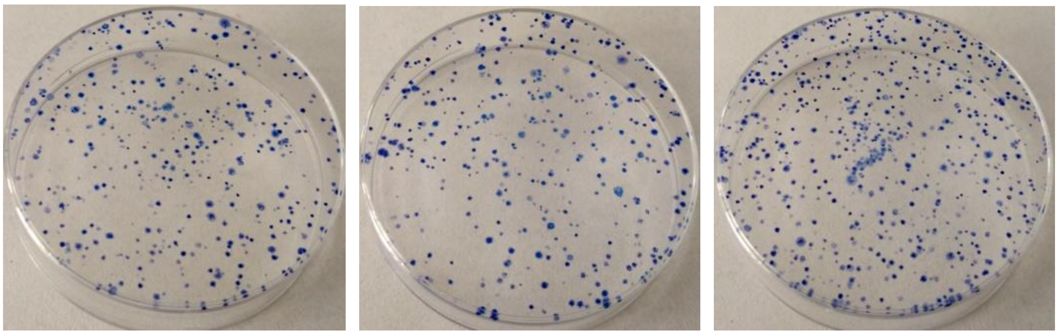

RKO

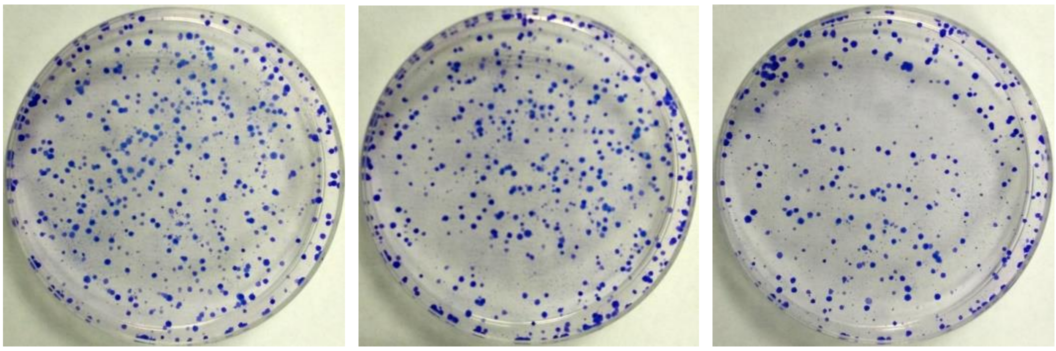

SW480
