# Supplementary material for: Dual roles of TRIM3 in colorectal cancer by retaining p53 in the cytoplasm to decrease its nuclear expression
Source: Cell Death Discov. 2023 Mar 9;9:85. doi: 10.1038/s41420-023-01386-1 (PMC9998637; doi:10.1038/s41420-023-01386-1)
Supplement: Supplementary file 7 — Figure 3-Original Data [file 41420_2023_1386_MOESM7_ESM.pdf]

Figure 3A

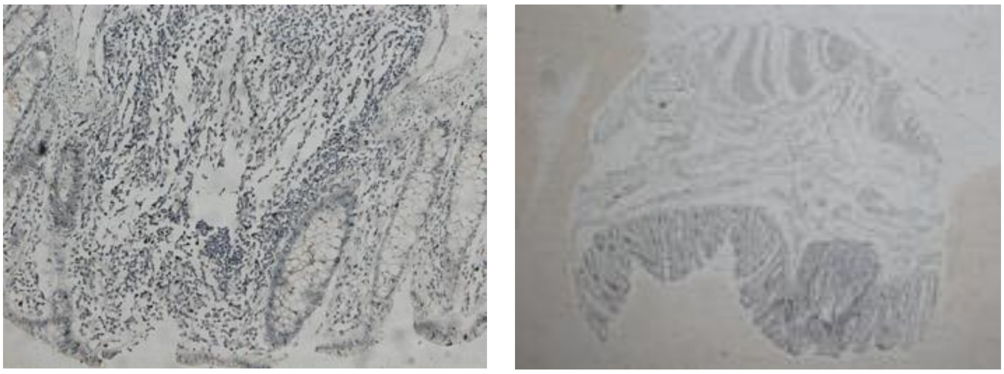

Normal-TRIM3

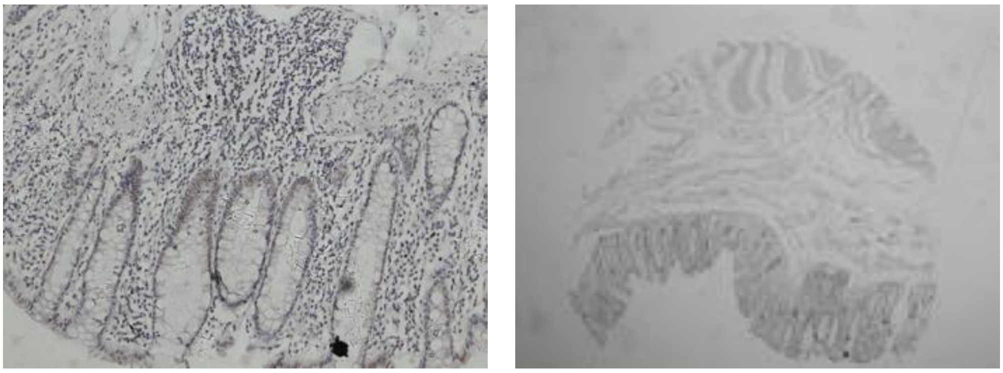

Normal-P53

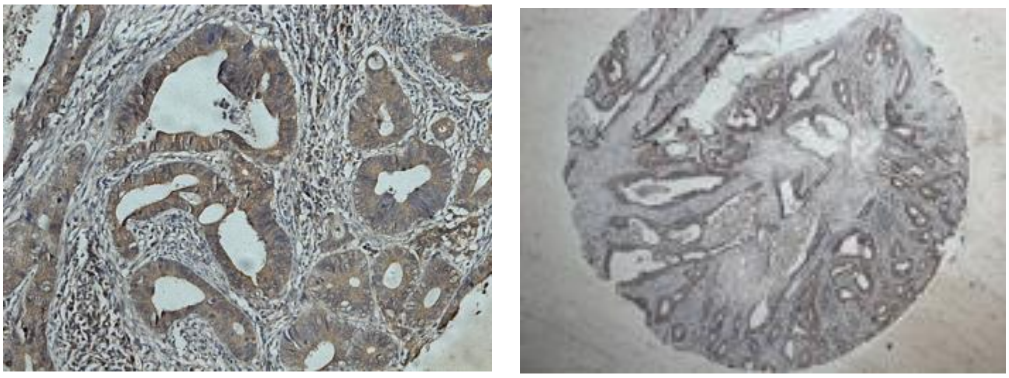

Well-differentiated-TRIM3

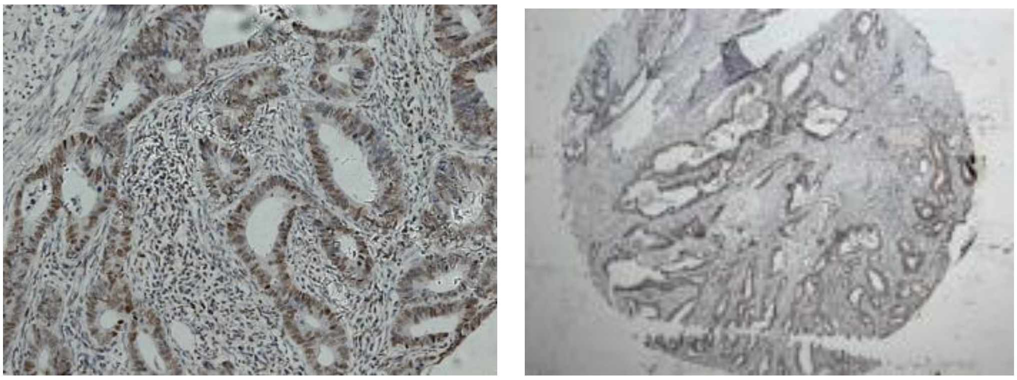

Well-differentiated-P53

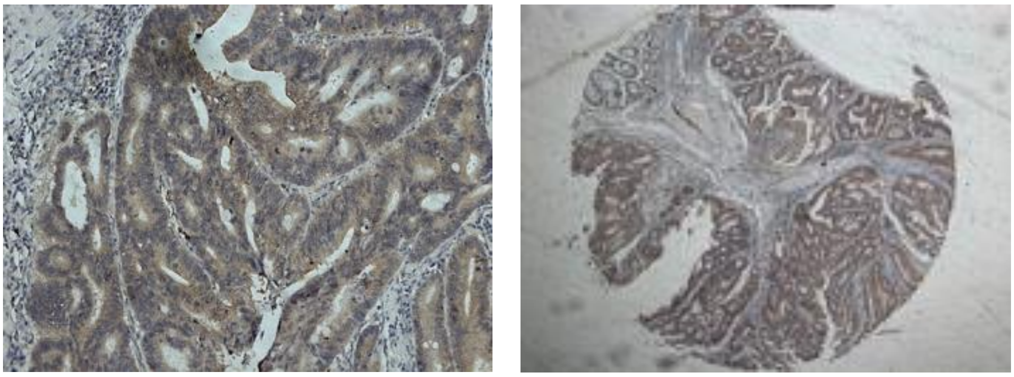

Moderately-differentiated-TRIM3

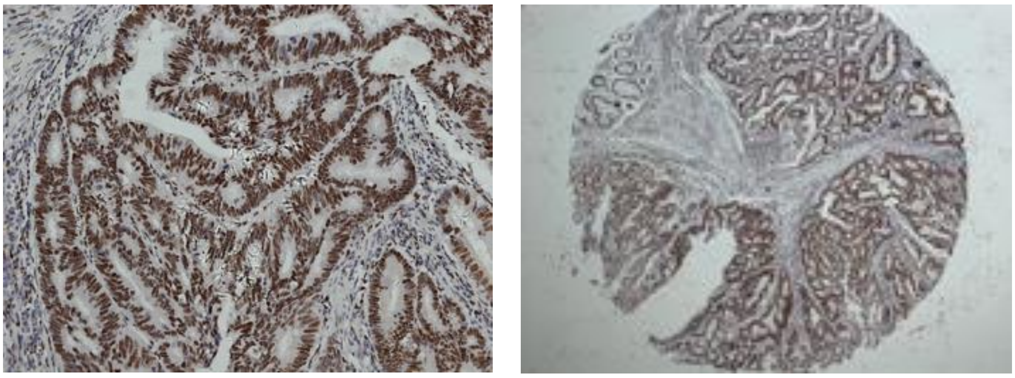

Moderately-differentiated-P53

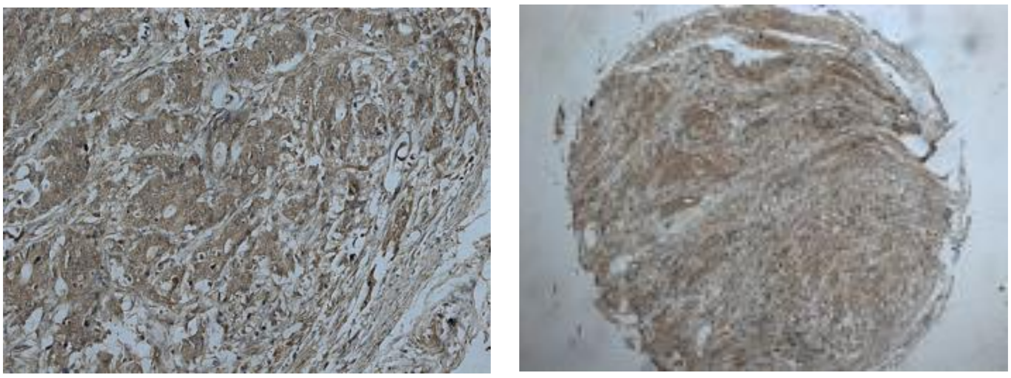

Poor-differentiated-TRIM3

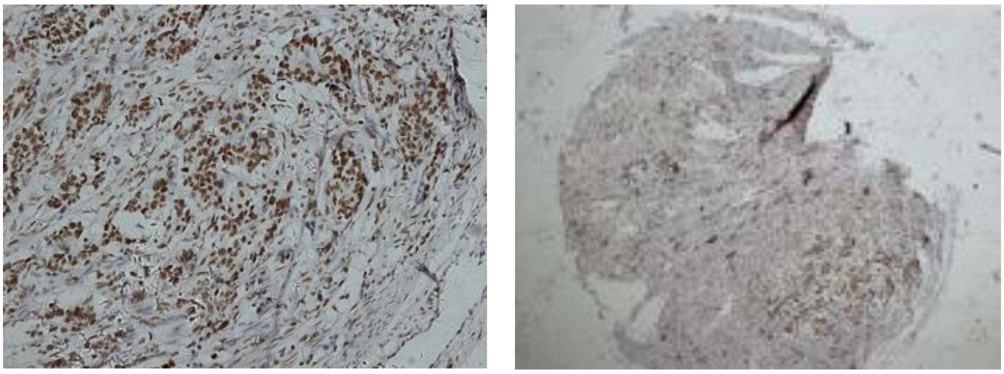

Poor-differentiated-P53

Figure 3B

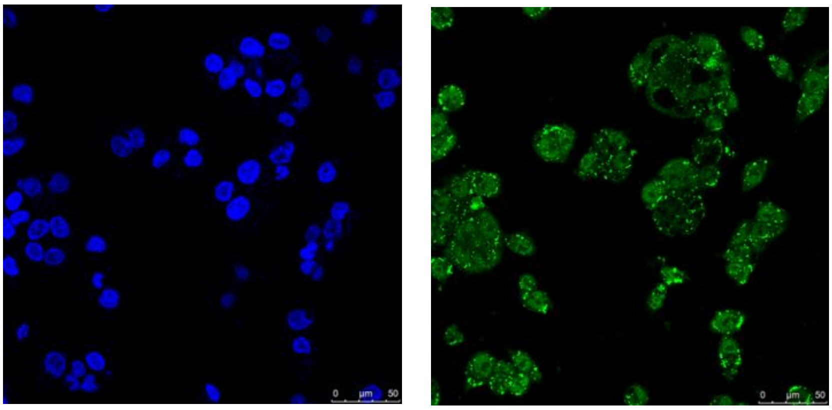

RKO-CON

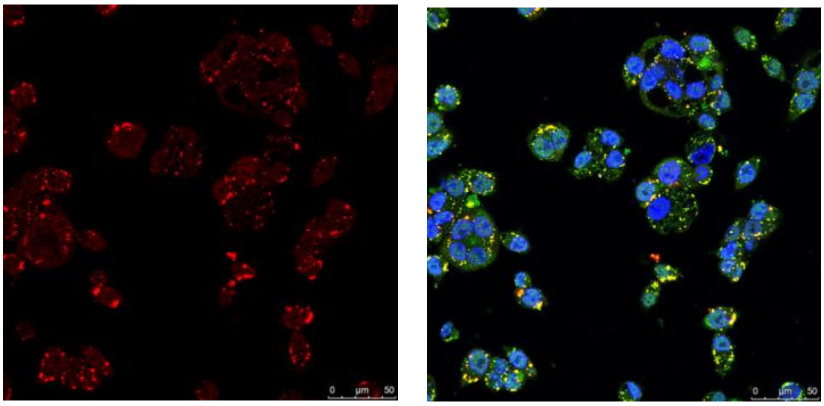

RKO-CON

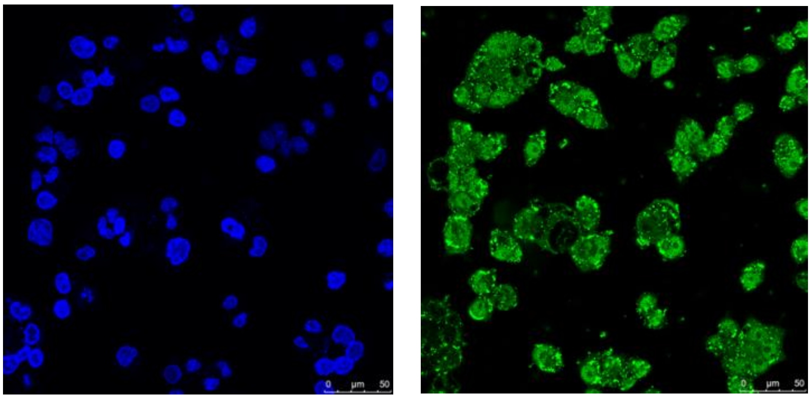

RKO-TRIM3

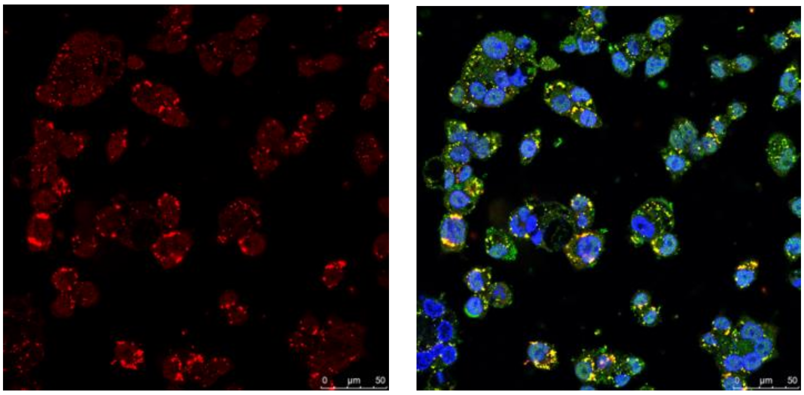

RKO-TRIM3

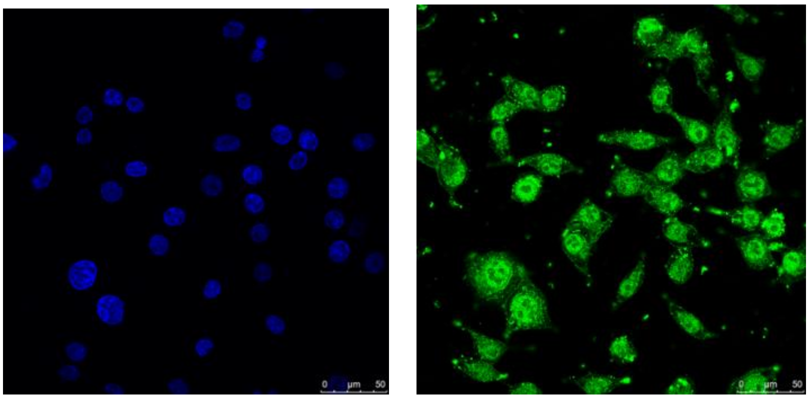

SW480-CON

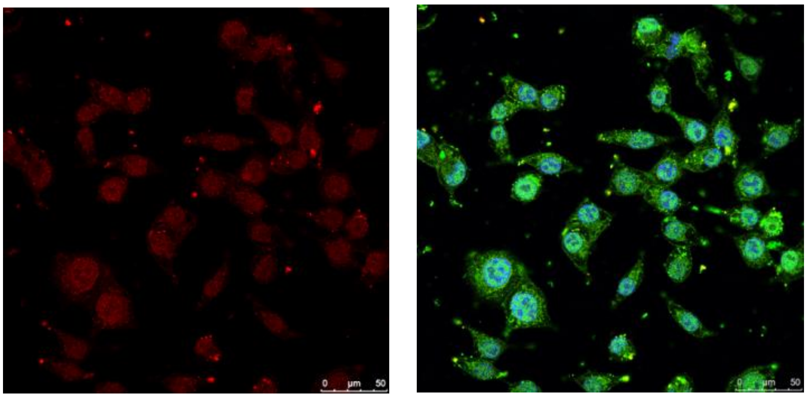

SW480-CON

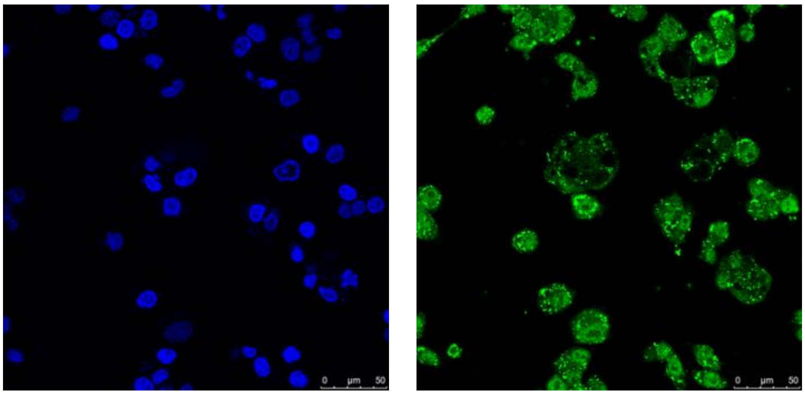

SW480-TRIM3

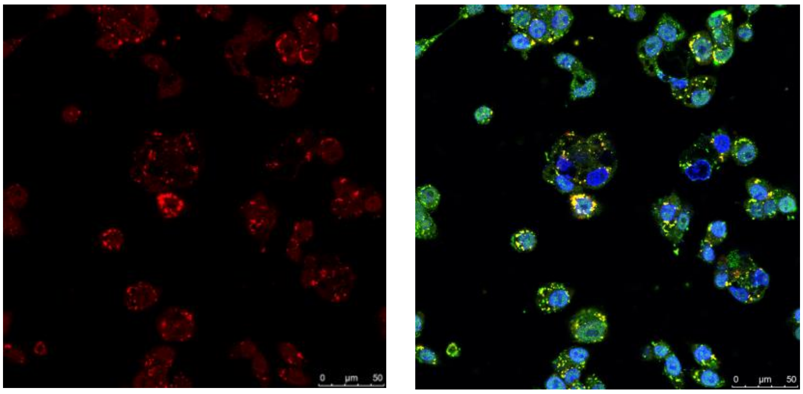

SW480-TRIM3

Figure 3C

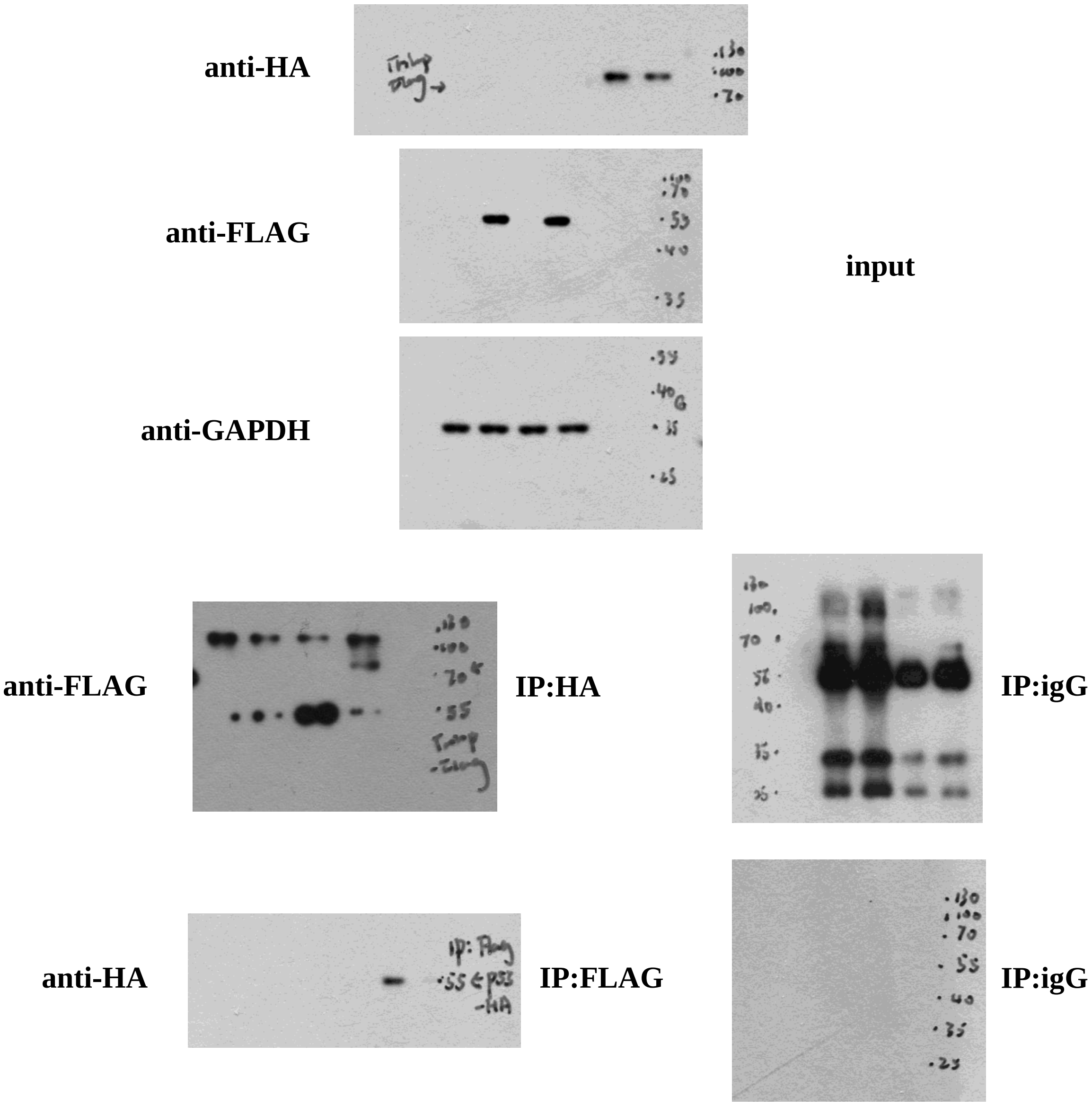

Figure 3E

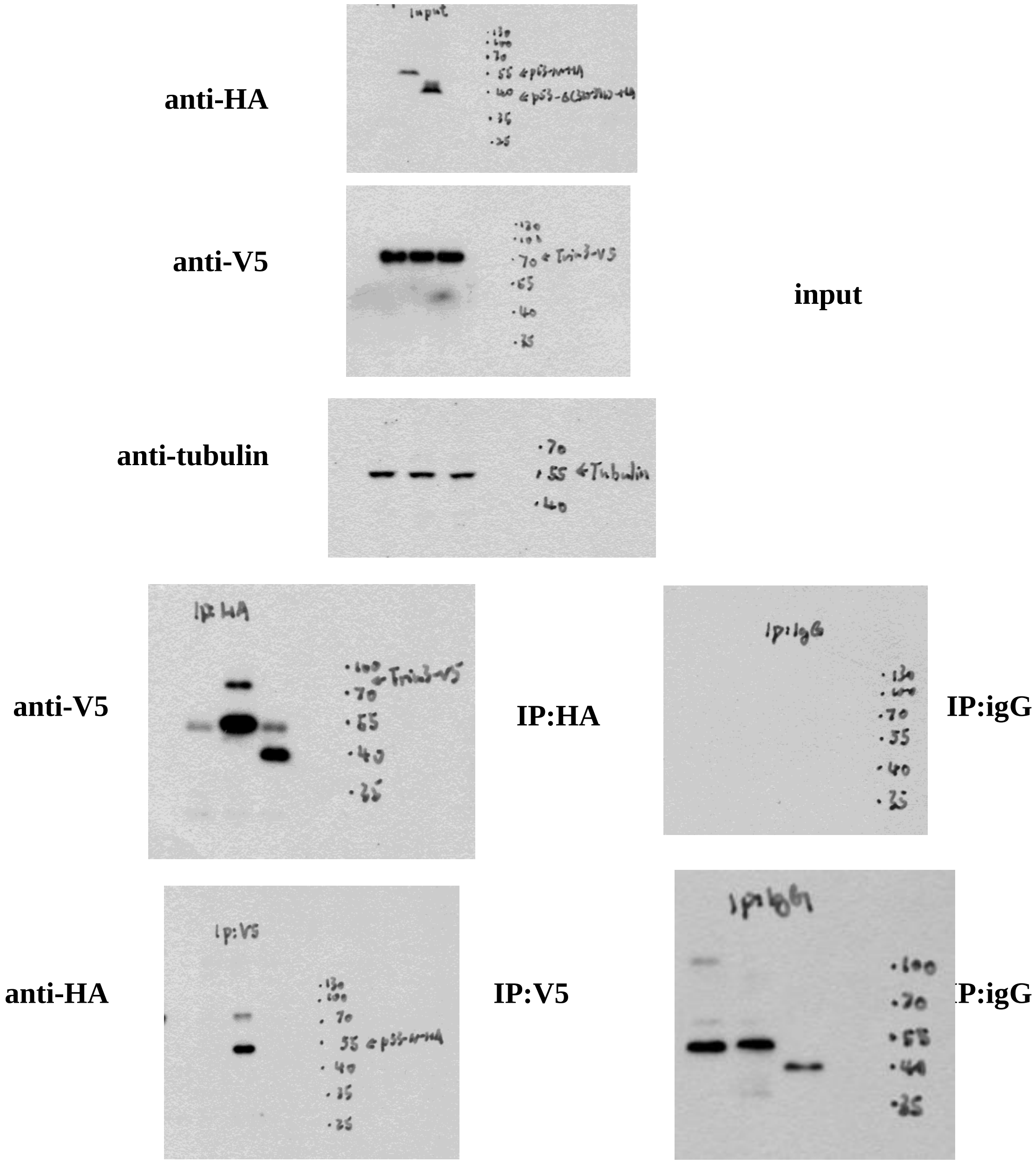
anti-HA
anti-V5
input
anti-tubulin
anti-V5
IP:HA
IP:igG
anti-HA
IP:V5
IP:igG
