# Supplementary material for: Dual roles of TRIM3 in colorectal cancer by retaining p53 in the cytoplasm to decrease its nuclear expression
Source: Cell Death Discov. 2023 Mar 9;9:85. doi: 10.1038/s41420-023-01386-1 (PMC9998637; doi:10.1038/s41420-023-01386-1)
Supplement: Supplementary file 8 — Figure 4-Original Data [file 41420_2023_1386_MOESM8_ESM.pdf]

Figure 4B1

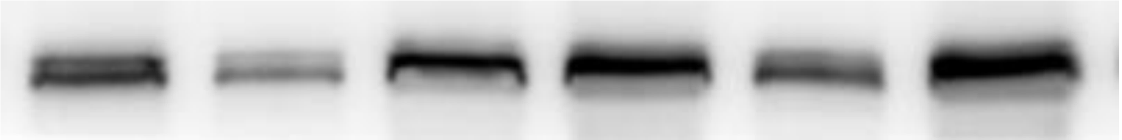

RKO-TRIM3

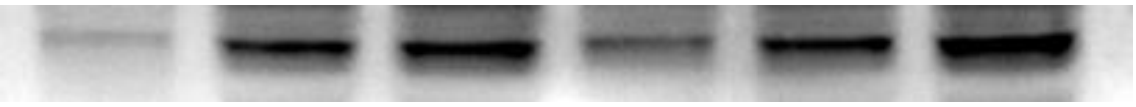

RKO-P53

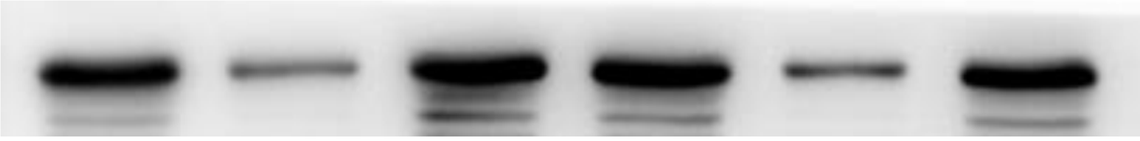

RKO-GAPDH

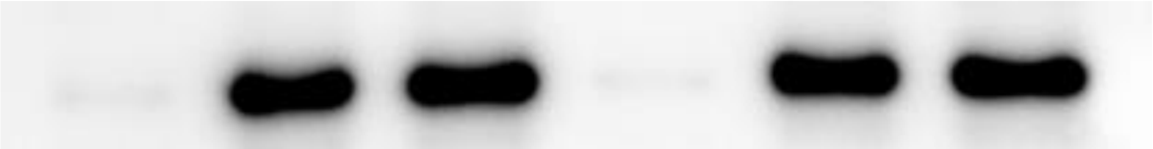

RKO-H3

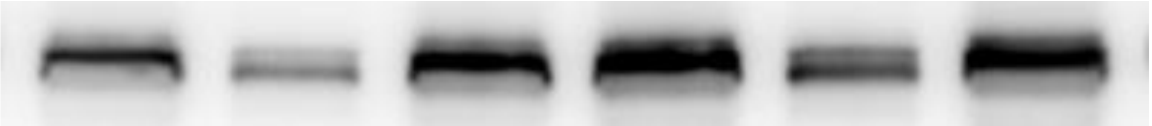

SW480-TRIM3

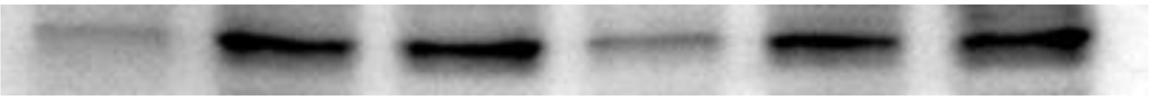

SW480-P53

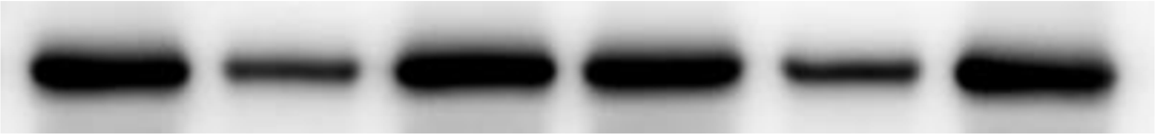

SW480-GAPDH

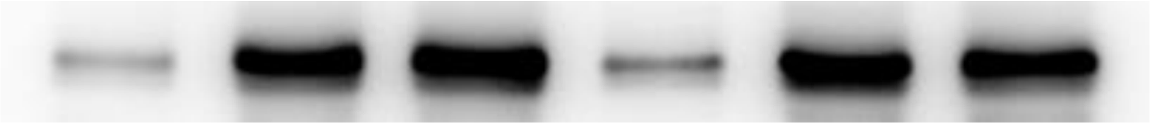

SW480-H3

Figure 4C

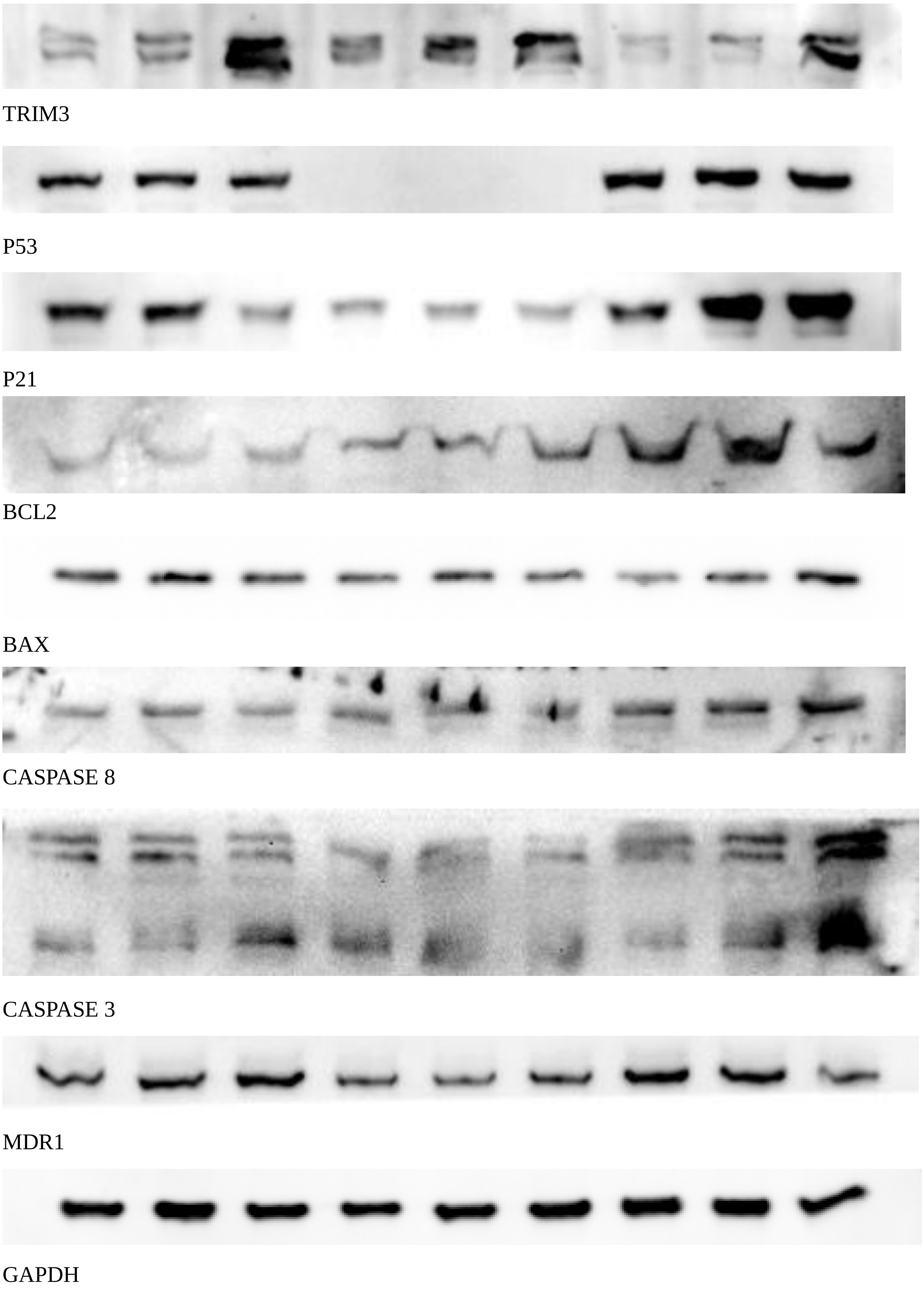

# Figure 4E

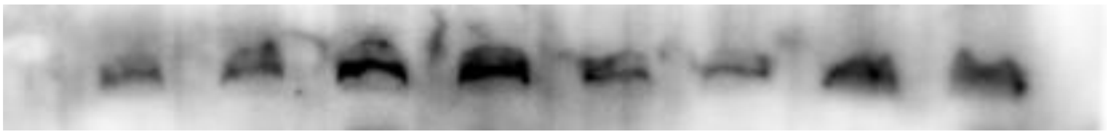

TRIM3

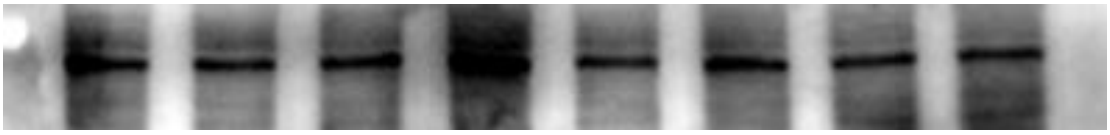

P53

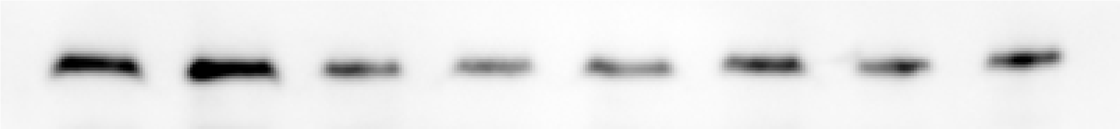

P21

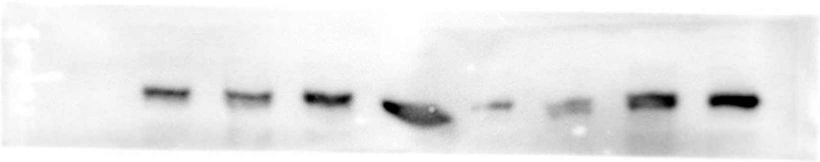

CASPASE 3

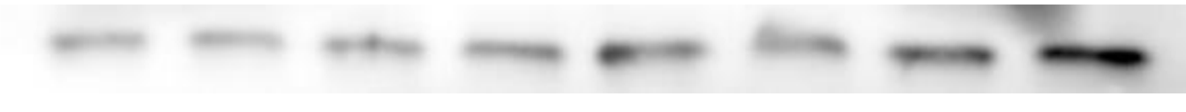

CASPASE 8

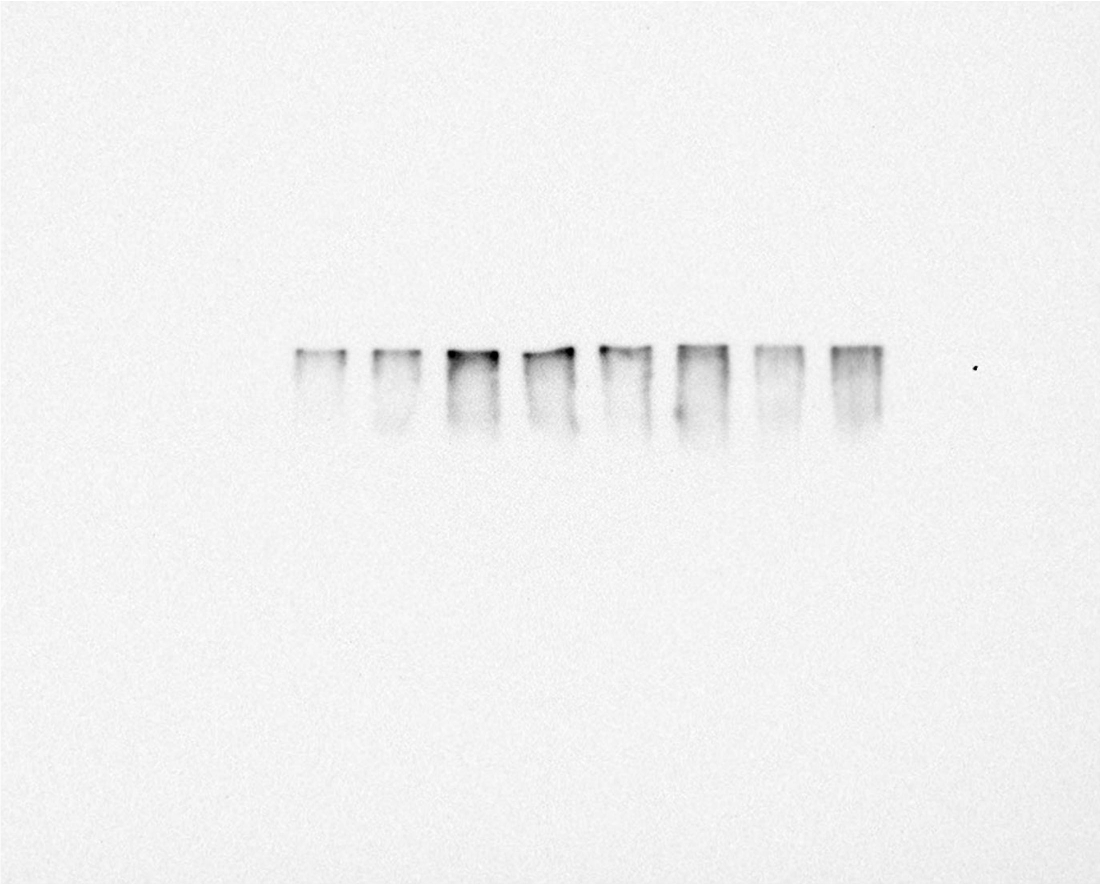

MDR1

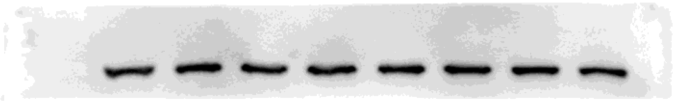

GAPDH
