# Supplementary material for: Dual roles of TRIM3 in colorectal cancer by retaining p53 in the cytoplasm to decrease its nuclear expression
Source: Cell Death Discov. 2023 Mar 9;9:85. doi: 10.1038/s41420-023-01386-1 (PMC9998637; doi:10.1038/s41420-023-01386-1)
Supplement: Supplementary file 9 — Figure 5-Original Data [file 41420_2023_1386_MOESM9_ESM.pdf]

Figure 5A1

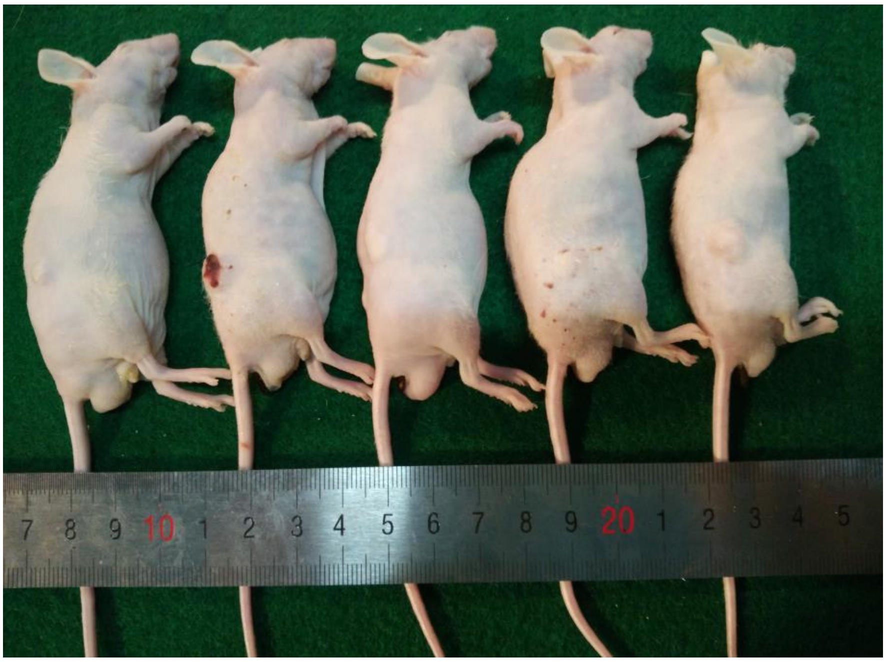

RKO-CON

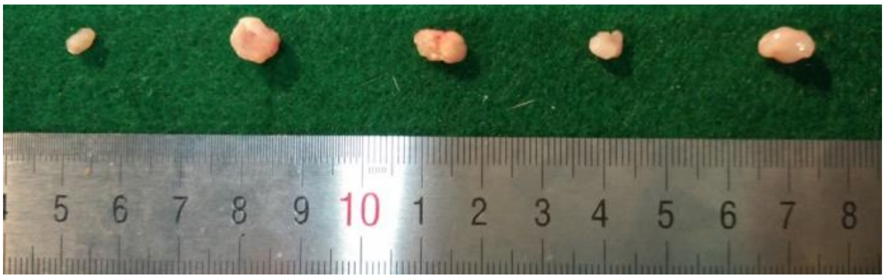

RKO-CON

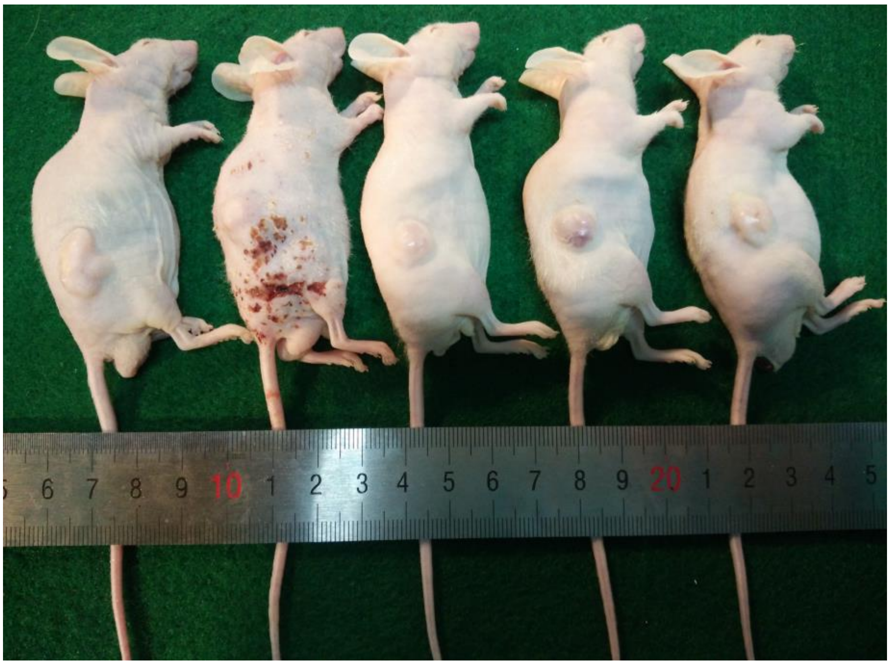

RKO-TRIM3

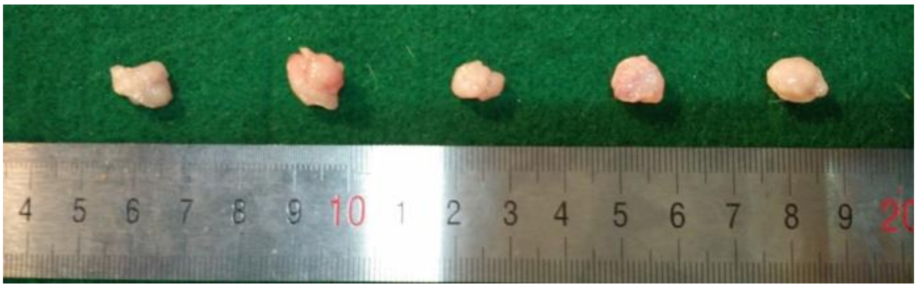

RKO-TRIM3

Figure 5B1

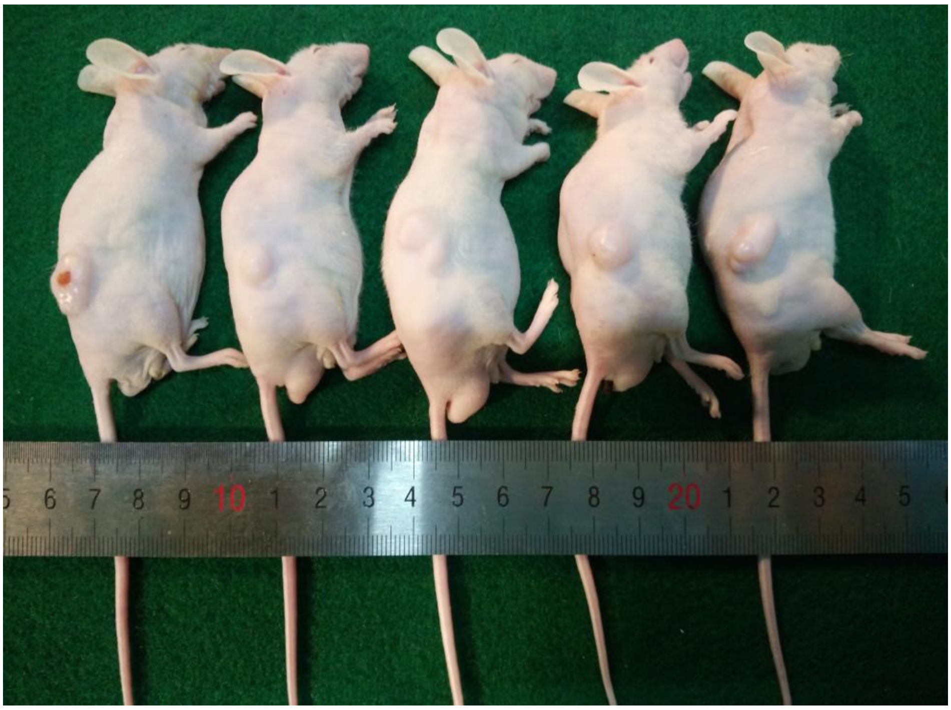

SW480-CON

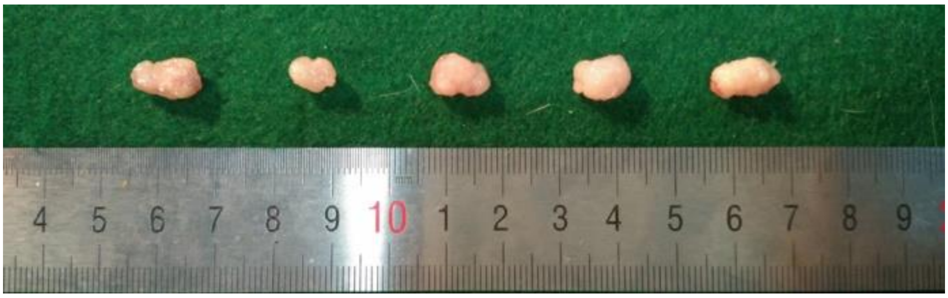

SW480-CON

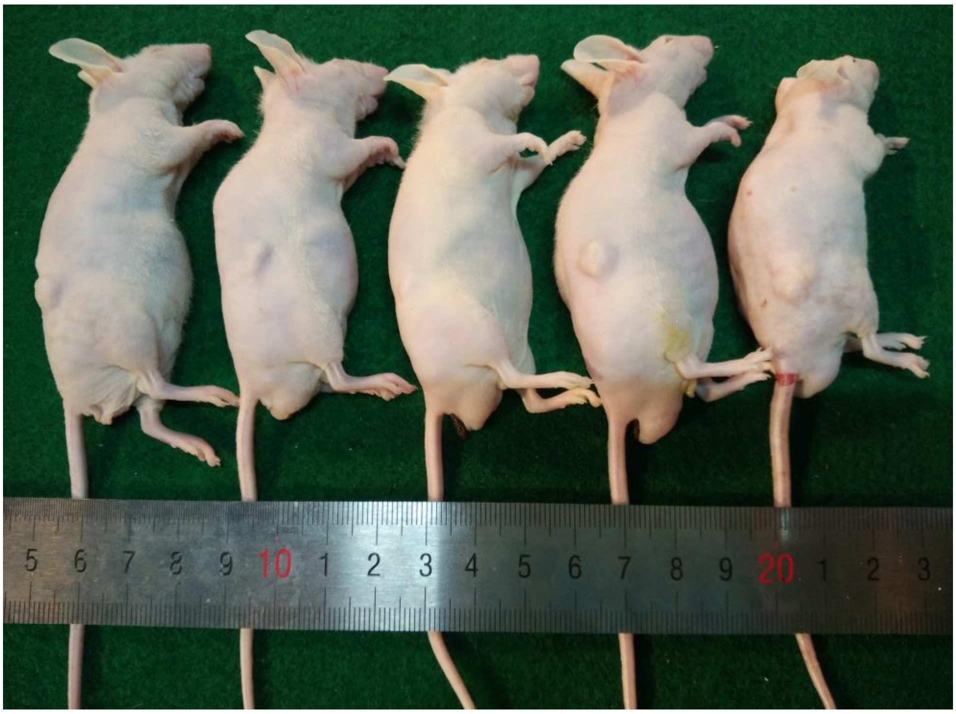

SW480-TRIM3

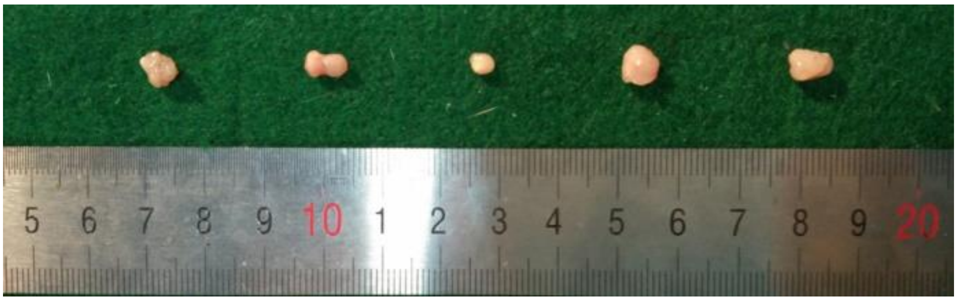

SW480-CON

Figure 5C1

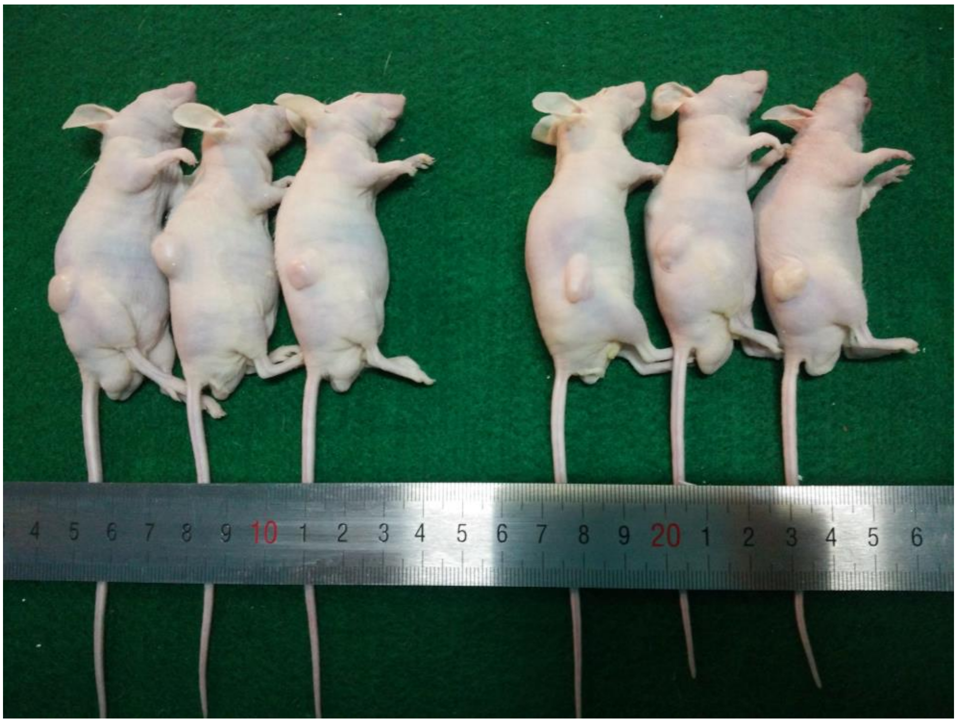

RKO-OX(treatment)

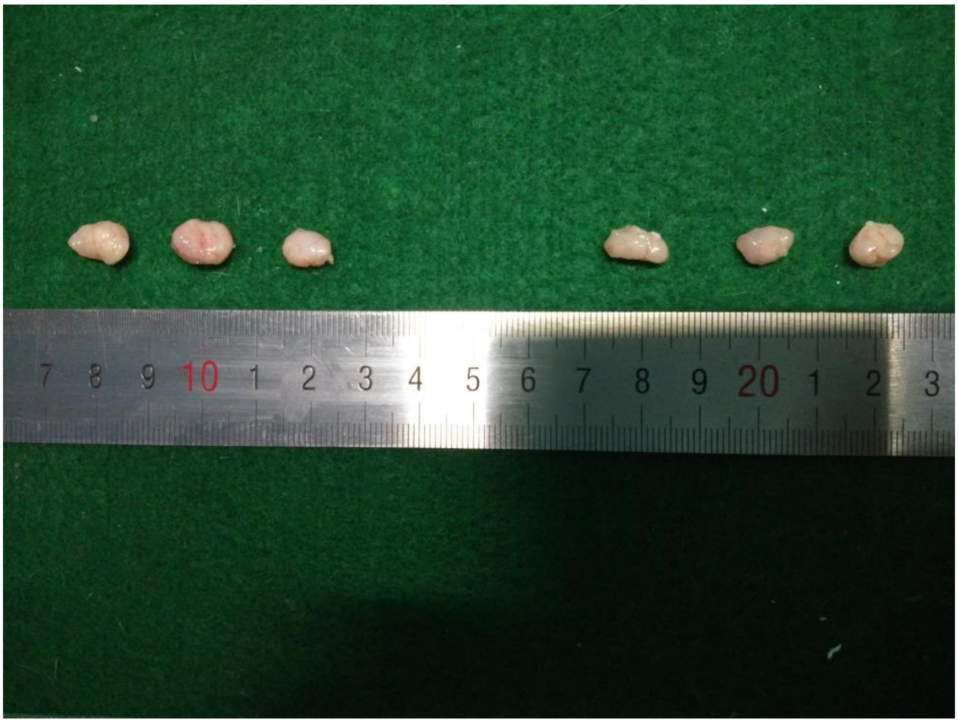

RKO-OX(treatment)

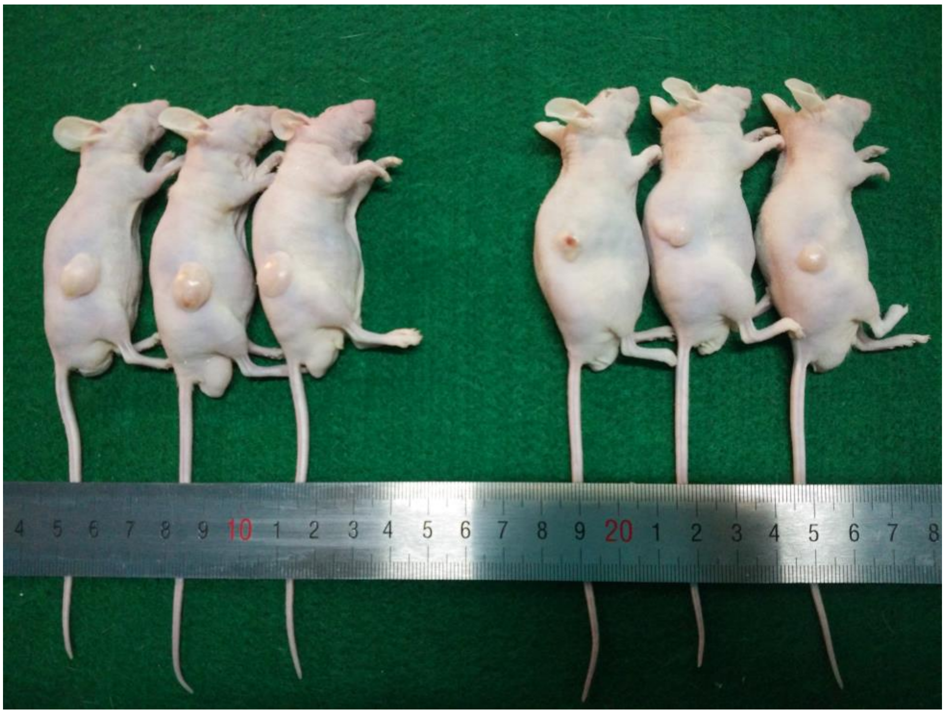

SW480-OX(treatment)

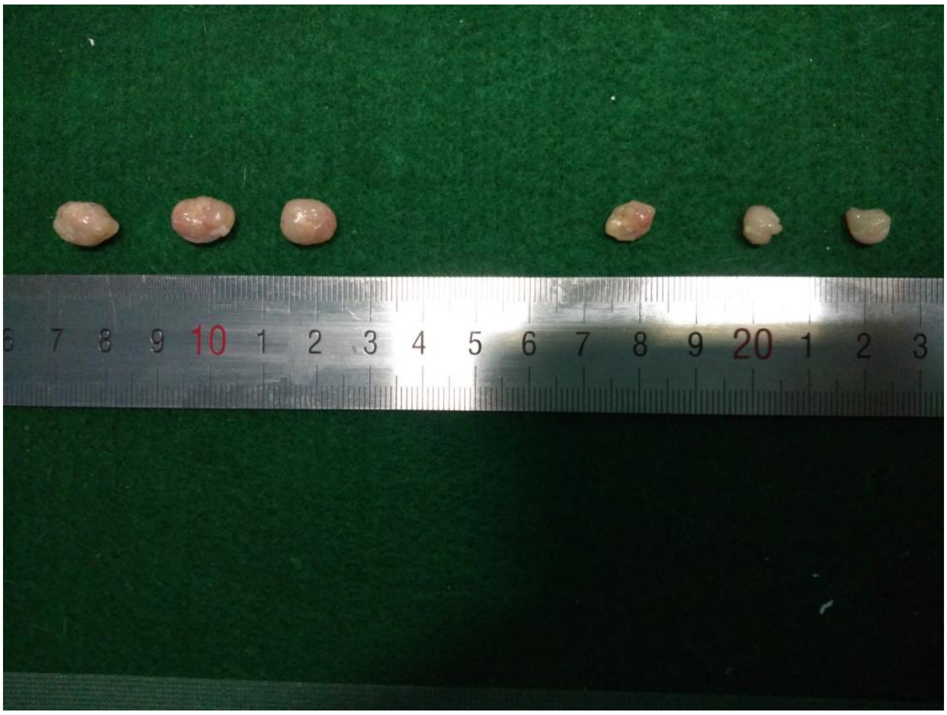

SW480-OX(treatment)
